# Effect of minimal intervention on carious lesions in primary teeth. An Umbrella review

**DOI:** 10.3389/fdmed.2025.1751752

**Published:** 2026-01-12

**Authors:** Tania Carola Padilla-Cáceres, Heber Isac Arbildo-Vega, Vilma Mamani-Cori, Luz Marina Caballero-Apaza, Fredy Hugo Cruzado-Oliva, Carlos Alberto Farje-Gallardo, Consuelo Marroquín-Soto, Rubén Aguirre-Ipenza, Hernán Vásquez-Rodrigo, Sara Antonieta Luján-Valencia, Joan Manuel Meza-Málaga, Tania Belú Castillo-Cornock, Franz Tito Coronel-Zubiate

**Affiliations:** 1 Department of General Dentistry, Dentistry School, Universidad Nacional del Altiplano, Puno, Peru; 2 Faculty of Dentistry, Dentistry School, Universidad San Martín de Porres, Chiclayo, Peru; 3 Faculty of Human Medicine, Human Medicine School, Universidad San Martín de Porres, Chiclayo, Peru; 4 Faculty of Post Graduate, Universidad Nacional Toribio Rodríguez de Mendoza de Amazonas, Chachapoyas, Peru; 5 Department of Nursing, School of Nursing, Universidad Nacional del Altiplano, Puno, Peru; 6 Faculty of Stomatology, Stomatology School, Universidad Nacional de Trujillo, Trujillo, Peru; 7 Faculty of Health Sciences, Universidad Nacional Toribio Rodríguez de Mendoza de Amazonas, Chachapoyas, Peru; 8 Department of Dentistry, School of Dentistry, Universidad Científica del Sur, Lima, Peru; 9 Faculty of Health Sciences, Universidad Continental, Lima, Peru; 10 Faculty of Health Sciences, Dentistry School, Universidad Norbert Wiener, Lima, Peru; 11 Postgraduate School, Universidad Católica de Santa María, Arequipa, Peru; 12 Faculty of Dentistry, Dentistry School, Universidad Católica de Santa María, Arequipa, Peru

**Keywords:** children, cognition, cognitive development, iron supplement, review

## Abstract

**Aim:**

To assess the effect of minimal intervention on carious lesions in primary teeth through an umbrella review.

**Material and methods:**

A comprehensive search was conducted in databases such as PubMed, Cochrane Library, Scopus, Web of Science, Embase, SciELO, Google Scholar, ProQuest Dissertations and Theses, and OpenGrey, covering literature up to September 2025. Systematic reviews, with or without meta-analyses, that assessed the effect of minimal intervention on carious lesions in primary teeth were included. Narrative reviews, rapid reviews, clinical trials, observational or experimental studies, case reports, editorials, letters, protocols, and posters were excluded. The methodological quality of the reviews was assessed using the AMSTAR-2 tool, and the risk of bias was assessed using the ROBIS tool.

**Results:**

From an initial retrieval of 498 records, 49 systematic reviews met the inclusion criteria. The data showed an impact of minimally invasive interventions on carious lesions in primary teeth.

**Conclusion:**

Based on high-confidence systematic reviews, among the minimally invasive interventions, the use of silver diamine fluoride, the Hall technique, and resin infiltration showed significant benefits for managing carious lesions in primary teeth.

**Systematic Review Registration:**

https://doi.org/10.17605/OSF.IO/3T2GV, Open Science Framework (OSF): doi: 10.17605/OSF.IO/3T2GV

## 1 Introduction

Dental caries in primary dentition remains the most prevalent chronic disease of childhood worldwide, affecting nearly half of preschool-aged children and exceeding 70% in populations with high social and economic risk (1–3). These injuries not only cause pain and functional impairment but also negatively impact nutrition, growth, and quality of life, representing a major global public health burden (4). In this context, early detection and timely intervention in deciduous teeth are essential to reduce disease progression and preserve children's oral health (1, 2).

In contrast to traditional restorative approaches focused on complete removal of decayed tissue, Minimal Intervention Dentistry (MID) has emerged as a preventive and conservative model grounded in the biological understanding of caries (5, 6). This philosophy aims to limit operative trauma through early detection, enamel remineralization, and selective tissue repair, prioritizing chemical disinfection and biofilm control over extensive restorations (7, 8). In primary dentition—where caries progresses rapidly and exfoliation is imminent—MID seeks to maintain pulpal integrity and extend tooth functionality without compromising aesthetics or child comfort (9). The American Dental Association (ADA) Clinical Practice Guideline supports this approach, recommending the sealing of active lesions and the use of topical fluorides as first-line therapy (10).

The available scientific evidence mainly derives from systematic reviews that have evaluated different minimally invasive interventions, such as silver diamine fluoride (SDF), sealants, the Hall technique, atraumatic restorative treatment (ART), resin infiltration, and laser-based methods (5, 11). However, the quality, methodological rigor, and certainty of these reviews are highly variable. Differences in eligibility criteria, outcome definitions (e.g., caries arrest, restoration survival, pulp exposure), follow-up periods, and risk-of-bias assessment have resulted in heterogeneous findings and, in some cases, conflicting conclusions, limiting their direct applicability to clinical decision-making (12–14).

Among the most studied alternatives, 38% SDF has shown particularly favorable results, achieving caries arrest rates ranging from 53% to 91%, depending on application frequency (4, 5, 15, 16). Despite its main drawback—irreversible black staining—SDF remains a highly effective, economical, and well-tolerated option in community and school settings (17). Fluoride varnishes (NaF 5%) also represent accessible interventions, demonstrating recovery of 15%–25% of incipient lesions following quarterly or four-monthly application schedules (10, 18, 19).

Fissure sealants continue to be a well-established preventive strategy, reducing caries risk by up to 75% over 24 months of follow-up, although their effectiveness is lower in primary molars and in lesions that have progressed into dentin (5, 20, 21). Effectiveness increases when combined with pretreatment using sodium fluoride or remineralizing agents such as casein phosphopeptide–amorphous calcium phosphate (22). Meanwhile, ART—based on high-viscosity glass ionomer cement—has demonstrated success rates of 78%–86% in single-surface cavities, showing comparable results to conventional restorations and high applicability in resource-limited contexts (5, 23–25).

Similarly, the Hall technique, which involves placing preformed stainless-steel crowns without tooth preparation or anesthesia, has reported 97% survival rates at two years and over 90% acceptance by both children and parents (26–28). Other microinvasive strategies, such as resin infiltration, have demonstrated a 43% reduction in radiographic progression of non-cavitated lesions (29–31), while chemical-mechanical caries removal with Papacarie® and selective caries excavation preserve the moist dentin near the pulp, reducing exposure risk and operative discomfort (32–36). Finally, dental lasers (Er:YAG, Er,Cr:YSGG, or diode 810–980 nm) have shown potential as painless adjunctive tools that enhance child cooperation and reduce the need for anesthesia, although clinical evidence remains limited (37, 38).

Taken together, these interventions reflect a clear transition toward a more biological and conservative approach focused on tissue preservation. However, the growing number of systematic reviews addressing overlapping minimally invasive strategies—often with different methodological quality, levels of confidence, and degrees of primary study overlap—has generated uncertainty regarding the true strength and consistency of the evidence. In this context, an umbrella review is particularly warranted to synthesize and critically appraise the existing body of systematic reviews, integrate assessments of methodological quality and risk of bias, and provide a hierarchical overview of the evidence supporting each intervention.

Therefore, the objective of this umbrella review is to evaluate the overall effect of minimal intervention approaches on carious lesions in primary teeth and to determine the confidence level of existing systematic reviews, thereby offering a comprehensive, evidence-based framework to support clinical decision-making and future research in pediatric dentistry.

## 2 Materials and methods

### 2.1 Protocol and registration

This umbrella review was conducted following the Preferred Reporting Items for Systematic Review and Meta-Analysis Protocols (PRISMA-P) guidelines (39), and was registered in the Open Science Framework (OSF) under the DOI number DOI 10.17605/OSF.IO/3T2GV (https://archive.org/details/osf-registrations-3t2gv-v1), which is publicly accessible. Furthermore, the reporting of this review adhered to the PRIO-harms checklist (Preferred Reporting Items for Overviews of Systematic Reviews) (40). Given the nature of the study, ethical approval was not required.

### 2.2 Eligibility criteria and results of interest

Studies eligible for inclusion were systematic reviews (with or without meta-analysis) assessing primary research determining the effect of minimal intervention on carious lesions in primary teeth. No restrictions were applied regarding publication date or language. Excluded publication types were narrative reviews, rapid reviews, interventional studies, observational research, preclinical and basic science studies, protocols, abstracts, case reports, commentaries, letters, opinions, and poster presentations.

For clarity, the research question was structured according to the PICOS framework. The population (P) comprised children with carious lesions in primary teeth. The interventions (I) included minimally invasive strategies such as silver diamine fluoride, atraumatic restorative treatment, sealants, resin infiltration, fluoride varnish, the Hall technique, chemical-mechanical caries removal, laser-assisted techniques, and selective caries excavation. The comparators (C) were conventional restorative or operative treatments, non-invasive management, placebo, or no treatment, as defined within the included systematic reviews. The outcomes (O) encompassed caries arrest or progression, restoration survival or failure, pulp exposure or pulpal vitality, need for retreatment, and absence of periapical pathology. The study designs (S) included systematic reviews, with or without meta-analyses, of primary clinical studies assessing these interventions in primary teeth.

### 2.3 Sources of information, search strategy and additional search for primary studies

An electronic literature search was conducted on September 25, 2025, using four major databases: PubMed, Cochrane Library, Scopus, Web of Science, Embase and SciELO. To identify additional records, gray literature sources were also consulted, including Google Scholar, ProQuest Dissertations and Theses, and OpenGrey. Reference lists of included studies were manually screened to identify any relevant additional publications. All retrieved articles were imported into Zotero® (Center for History and New Media, Virginia, USA), and duplicates were removed. Detailed search strategies for each database are presented in Supplementary Material S1.

### 2.4 Data management and selection process

The screening and selection process was carried out using Rayyan® online software (Qatar Computing Research Institute, Qatar). Study selection was conducted in two phases. In the first phase, two independent reviewers (L.C. and T.P.) assessed titles and abstracts. In the second phase, the full texts of potentially relevant studies were evaluated independently by the same reviewers. Disagreements at any stage were resolved through discussion with a third reviewer (H.A.).

### 2.5 Data collection process

Data extraction was carried out independently and in duplicate by two reviewers (V.M. and F.C.O.) using a standardized data collection form. Extracted information was cross-checked for consistency, and disagreements were resolved by a third author (H.A). The following variables were recorded: author names, year of publication, type of systematic review, characteristics of included primary studies, number of studies included in qualitative and quantitative analyses, reported outcomes, main conclusions, and whether the reviews reported adherence to PRISMA guidelines, PROSPERO registration or another public platform, use of the GRADE system, and performance of a meta-analysis.

### 2.6 Assessment of methodological quality, quality of evidence and meta-bias

The methodological quality and meta-bias or the risk of bias of the included systematic reviews was assessed independently and in duplicate by two reviewers (F.C.Z. and R.A.), calibrated (Kappa 0.85), using the AMSTAR-2 checklist (A Measurement Tool to Assess Systemic Reviews) (41) and the ROBIS tool (42). The overall confidence level in the studies was rated as high, moderate, low, or critically low, and the risk of bias was rated as high, unclear or low.

### 2.7 Summary of measures

For systematic reviews (SRs) that did not include a meta-analysis, the extracted outcomes were reported by determining the effect of minimal intervention on carious lesions in primary teeth. In cases where the SRs provided a meta-analysis, the results were recorded using either odds ratio (OR), risk ratio (RR), mean difference (MD) or weighted mean difference (WMD).

### 2.8 Summary of results

The primary findings from the included systematic reviews were organized and reported according to key outcomes, including ART, chemical-mechanical caries removal, fluoride varnish, Hall technique, laser, resin infiltration, SDF, sealant and selective excavation.

## 3 Results

### 3.1 Review and selection of primary studies

**Figure 1:**
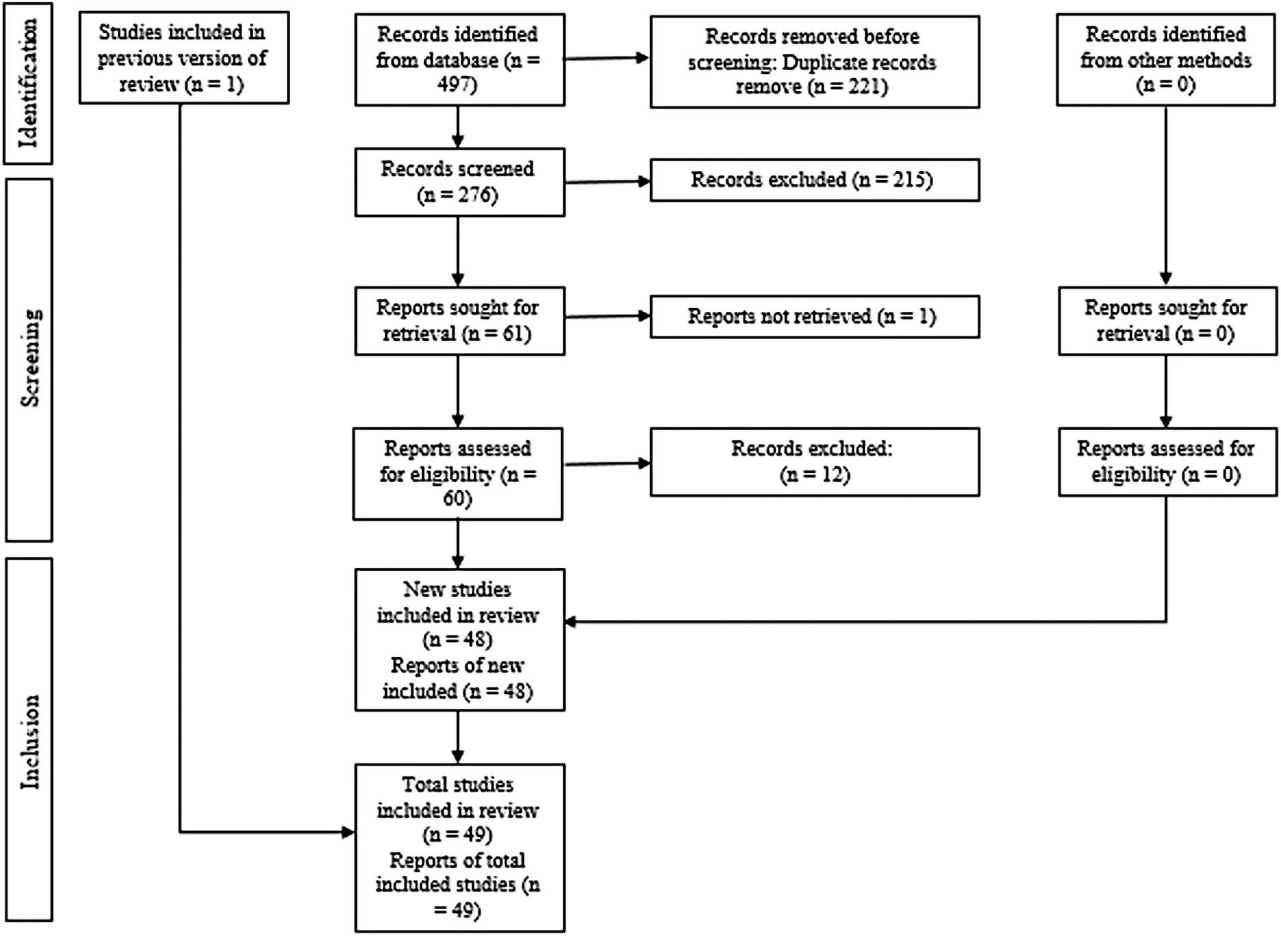
PRISMA flow diagram showing the selection process of studies included in the systematic review, from initial identification to final inclusion.

A total of 498 records were identified through the electronic database search. After eliminating duplicates, 277 unique references remained. In the initial screening phase, titles and abstracts were reviewed, resulting in 61 studies deemed suitable for full-text assessment. Of these, 12 studies were excluded, leaving 49 studies for analysis. Details regarding the exclusion criteria applied during the selection process are provided in Supplementary Material S2. The complete workflow for study identification and selection is illustrated in Figure 1.

### 3.2 Review and characteristics of included studies

The included SRs were published between 2006 and 2025. The studies originated from a diverse range of countries, including Italy (23, 28, 38, 43–46), India (47–49), Saudi Arabia (50–54), Pakistan (50), Romania (43), United Kingdom (27, 43, 44, 55–63), United State (16, 52, 56, 58, 64, 65), Singapore (57, 66), Qatar (66), Germany (57–60, 64, 67, 68), Malaysia (55), Chile (27, 55), Brazil (9, 15, 16, 56, 69–73), Denmark (58), China (33, 74–79), France (80), Switzerland (9, 81), Australia (76), Spain (27), Puerto Rico (82), Greece (81), Austria (81), Netherlands (70, 73, 83) and Japan (78).

The evidence base comprised both systematic reviews with meta-analysis and narrative syntheses, and was mainly underpinned by randomized clinical trials. Across reviews, the most frequently evaluated minimal intervention approaches were silver diamine fluoride (SDF), the Hall technique (HT), atraumatic restorative treatment (ART), resin infiltration, sealants, selective caries removal/partial excavation, chemical-mechanical caries removal (e.g., Papacarie/Carisolv), fluoride varnish, and lasers.

In terms of outcomes, the included SRs predominantly reported clinical endpoints relevant to pediatric caries management, such as caries arrest/progression, restoration survival/failure, treatment success rates, and—when available—pulp-related outcomes (e.g., pulp exposure, symptoms, vitality) and complications during follow-up. A subset of reviews also reported patient-centered or procedural outcomes (e.g., discomfort/pain during treatment) and feasibility aspects (e.g., suitability for community or resource-limited settings). Further details regarding the full characteristics and outcomes reported in each SR are provided in Supplementary Material S3.

### 3.3 Assessment of methodological quality, quality of evidence and meta-bias

25 SRs were considered to have high confidence (9, 15, 27, 28, 43–45, 47, 50, 52, 53, 55–64, 66, 67, 70, 81), 2 SRs had moderate confidence (23, 51), 13 SRs they had low confidence (16, 38, 48, 49, 65, 68, 74, 75, 77–80, 82) and 9 SRs had critically low confidence (33, 46, 54, 69, 71–73, 76, 83) (Supplementary Material S4). Furthermore, 24 SRs were found to have a high risk of bias (16, 23, 33, 38, 46, 48, 49, 51, 54, 65, 68, 69, 71–80, 82, 83) and 25 SRs were found to have a low risk of bias (9, 15, 27, 28, 43–45, 47, 50, 52, 53, 55–64, 66, 67, 70, 81) (Supplementary Material S3).

To mitigate the impact of low-confidence reviews on our overall findings, a sensitivity analysis was conducted, excluding studies with low, moderate or critically low confidence, and high risk of bias. The results presented here are based exclusively on high-confidence and low risk of bias reviews, ensuring the reliability of our conclusions.

Overall, the methodological certainty across reviews was mixed: approximately half of the included SRs were rated as high confidence by AMSTAR-2, whereas the remainder showed limitations leading to moderate-to-critically low confidence. ROBIS judgments followed a similar pattern, with roughly half of the SRs judged as low risk of bias and the other half as high risk of bias. These findings indicate that conclusions should be driven primarily by SRs with high overall confidence and low risk of bias, and that results from lower-quality SRs should be interpreted cautiously. Accordingly, our main conclusions emphasize evidence derived from high-confidence/low-risk SRs, while the full AMSTAR-2 item-level judgments and ROBIS assessments for each SR remain available in Supplementary Materials S3 and S4.

### 3.4 Overlapping

A total of 634 primary studies were identified within the SRs. The degree of overlap according to the CCA index is 1.45%, and this value indicates “slight overlap”. Specifically, 62 studies were included twice, 31 appeared three times, 13 were included four times, 5 appeared five times, 6 were included six times, 3 appeared seven times, 3 were included eight times and 1 study were included nine times. Further details on the degree of overlap and characteristics of the primary studies are provided in Supplementary Material S5. This slight overlap reduces the likelihood that our conclusions are driven by redundant inclusion of the same primary studies across multiple SRs, thereby supporting the independence of the evidence mapped in this umbrella review.

### 3.5 Synthesis of results

The synthesis of the results is presented in Supplementary Material S3.

### 3.6 ART

Ten SRs (9, 23, 27, 48, 57, 70, 71, 73, 76, 83) evaluated the effect of ART in primary teeth. Overall, ART showed either similar or superior effectiveness compared with conventional treatments. Five SRs reported comparable outcomes, whereas six SRs indicated better performance of ART, particularly in single-surface cavities.

Five SRs (9, 27, 57, 70, 71) meta-analyzed the results, where they found that the OR ranged from 0.89 [CI: 0.57–1.37] (70) to 1.60 [CI: 1.13–2.27] (27) and the RR ranged from 0.98 [CI: 0.94–1.04] (9) to 1.49 [CI: 1.15–1.93] (57).

The superiority of ART reported in these reviews was mainly associated with clinical outcomes such as restoration survival, caries control, and reduced operative invasiveness, particularly in single-surface cavities. Reviews indicating better performance emphasized comparable or higher restoration longevity, effective caries arrest using high-viscosity glass ionomer cement, and a lower risk of pulpal complications due to selective tissue removal. These advantages are especially relevant in pediatric patients, as ART minimizes operative trauma, reduces the need for local anesthesia, and improves child cooperation without compromising clinical effectiveness.

### 3.7 Chemical-mechanical caries removal

Three SRs (33, 46, 77) evaluated chemical-mechanical caries removal in primary teeth. Two reported better performance compared with conventional treatments, while one found similar effects. Meta-analyses reported an MD of 0.57 [CI: 0.04–1.09] (33) and an OR of 0.33 [CI: 0.01–8.22] (46).

The improved performance of chemical-mechanical caries removal reported in these reviews was primarily related to reduced operative trauma, improved child cooperation, and preservation of sound dentin, rather than superior restoration longevity. These outcomes are particularly relevant in pediatric patients, where minimizing discomfort and the risk of pulp exposure is a key clinical objective.

### 3.8 SDF

Fifteen SRs (9, 15, 16, 43, 44, 47, 49, 51–53, 65, 77–79, 82) evaluated SDF in primary teeth. Thirteen SRs reported superior performance compared with conventional treatments, one reported similar effects, and one favored conventional treatments. Seven SRs conducted meta-analyses, reporting effect estimates ranging from MD −0.27 [CI: −0.46 to −0.07] (51) to OR 18.10 [CI: 3.89–84.15] (53), with RR values between 1.11 [CI: 1.03–1.20] (9) and 2.54 [CI: 1.67–3.85] (15).

Across the included reviews, the superiority of SDF was consistently associated with higher caries arrest rates and reduced lesion progression, rather than restorative outcomes. These effects were most pronounced in cavitated lesions and in community-based or non-invasive care settings, highlighting SDF's effectiveness as a caries management strategy rather than a conventional restorative intervention.

### 3.9 Fluoride varnish

Two SRs (56, 62) reported that fluoride varnish showed similar effects to conventional treatments in primary teeth. Meta-analyses indicated OR values ranging from 0.99 [CI: 0.10–10.00] to 2.10 [CI: 0.52–9.00] (56), and an RR of 0.81 [CI: 0.62–1.06] (62).

Findings were consistent across lesion locations (proximal, occlusal, and buccal/lingual), suggesting comparable effectiveness rather than superiority over conventional approaches.

### 3.10 Hall technique

Six SRs (9, 28, 58, 59, 64, 66) evaluated the Hall technique in primary teeth. Four SRs reported superior performance compared with conventional treatments, while two reported similar outcomes. Meta-analyses reported OR values ranging from 1.06 [CI: 0.65–1.73] (66) to 8.35 [CI: 3.73–18.68] (58), and RR values from 0.18 [CI: 0.06–0.56] (59) to 1.30 [CI: 1.09–1.54] (9).

Overall, evidence favored the Hall technique, particularly for proximal lesions, with lower failure rates compared to conventional complete caries removal, although similar performance was observed when compared with alternative crown-based approaches.

### 3.11 Selective excavation

Nine SRs evaluated selective excavation in primary teeth. Five reported superior performance compared with conventional treatments, while four reported similar effects. Meta-analyses showed OR values ranging from 0.10 [CI: 0.04–0.25] (80) to 4.43 [CI: 1.04–18.77] (58), and an RR of 0.33 [CI: 0.01–7.62] (61).

The reported advantages of selective caries excavation were mainly related to a reduced risk of pulp exposure and maintenance of pulpal vitality, while achieving restoration survival comparable to conventional complete caries removal. This approach emphasizes biological preservation over mechanical completeness, which is particularly advantageous in primary teeth.

### 3.12 Laser

Two SRs (38, 45) evaluated laser use for carious lesions in primary teeth. One reported better performance, while the other found similar effects compared with conventional treatments. Meta-analysis results showed RR values ranging from 0.30 [CI: 0.11–0.78] to 1.00 [CI: 0.99–1.01], indicating no consistent superiority.

### 3.13 Resin infiltration

Six SRs (50, 54, 56, 60, 67, 74) reported superior performance of resin infiltration compared with conventional treatments in primary teeth. Meta-analyses reported OR values ranging from 0.15 [CI: 0.06–0.39] (60) to 6.90 [CI: 2.00–24.00] (56), with consistent benefits observed particularly in proximal non-cavitated lesions.

### 3.14 Sealant

Six SRs evaluated sealant use in primary teeth. Two reported superior performance, while four reported similar effects compared with conventional treatments. Meta-analyses showed OR values ranging from 0.23 [CI: 0.18–0.30] (74) to 5.00 [CI: 0.51–49.27] (58), and RR values between 0.67 [CI: 0.35–1.26] (38) and 1.06 [CI: 0.45–2.49] (81).

Evidence suggested greater effectiveness for proximal lesions, although overall findings indicated comparable rather than clearly superior outcomes.

## 4 Discussion

Dental caries in primary teeth continues to represent a major clinical and public health challenge, as it affects not only oral function and health in childhood but may also influence the eruption and health of the permanent dentition. From a research perspective, minimally invasive intervention approaches have emerged in pediatric dentistry with distinct procedure-specific clinical benefits. Techniques such as sealants and resin infiltration are primarily associated with arresting early non-cavitated lesions and delaying disease progression while preserving tooth structure. Chemical-mechanical caries removal and selective caries excavation focus on reducing operative trauma and lowering the risk of pulp exposure by preserving affected but firm dentin, thereby maintaining pulpal vitality during follow-up. Silver diamine fluoride (SDF) has been consistently linked to high caries arrest rates without operative intervention, making it particularly suitable for uncooperative children and community-based settings. The Hall technique, by sealing carious lesions without tissue removal, has demonstrated improved restoration longevity, reduced pulp exposure, and lower failure rates, with an absence of periapical pathology in longitudinal follow-up. Collectively, these approaches simplify clinical procedures, minimize trauma, and improve access to care, particularly in resource-limited settings. Importantly, the certainty of the evidence synthesized in this umbrella review is heterogeneous. Although a substantial proportion of the included systematic reviews were rated as high confidence with low risk of bias, a relevant number were classified as low or critically low confidence and/or high risk of bias. Therefore, the interpretation of findings in this Discussion prioritizes outcomes supported by systematic reviews with high AMSTAR-2 confidence and low ROBIS risk of bias, while results derived mainly from lower-quality reviews should be interpreted cautiously.

The results of this umbrella review, which included 49 systematic reviews on the management of carious lesions in primary teeth using minimally invasive strategies, show a low overlap of primary studies (1.45%). This low degree of overlap suggests that the different reviews provide relatively independent evidence and reinforces the uniqueness of the compiled findings. Of the 49 reviews included, 25 had high overall confidence, 2 moderate confidences, 13 low confidence, and 9 critically low confidences. In parallel, 24 reviews reported a high risk of bias and 25 a low risk of bias. These findings create a mixed picture regarding the robustness of the existing evidence, requiring cautious interpretation.

### 4.1 Evidence summary

Regarding ART, ten SRs assessed its effect in primary teeth; five of these had high confidence. Among those five, two concluded that ART performed better than conventional treatments, while three found similar outcomes. This aligns with a previous study (5), which defined ART success primarily as restoration survival without need for retreatment or pulpal complications, reporting higher success rates in single-surface cavities (86%) compared to multi-surface restorations, although the overall study quality was variable.

For chemo-mechanical caries removal, three SRs examined this approach in primary teeth, none with high confidence. Of these, two concluded that it performed better than conventional treatments, and one reported similar results. The observed advantage may be due to reduced trauma and greater tissue preservation, which improve child cooperation and reduce complications. Although promising, the lack of high-confidence reviews limits generalization.

Regarding SDF use in primary teeth, fifteen SRs addressed this intervention; seven had high confidence, and among these, six concluded that SDF performs better than conventional treatments, while one found similar results. This strengthens the high-quality evidence supporting SDF's strong caries-arresting effect in primary dentition (5), consistent with prior reviews suggesting that SDF is significantly more effective in halting caries progression compared to other treatments or placebo (4, 15).

For fluoride varnish, two SRs were identified, both with high confidence; all concluded that its performance is similar to conventional treatments in primary teeth. This suggests that although fluoride varnish is effective as a preventive or early-arrest measure, it does not clearly outperform other restorative or caries management techniques, particularly in advanced cavitations where its effect may be limited.

The Hall technique was examined in six SRs, all with high confidence: four reported better performances than conventional methods, and two found similar results. This agrees with evidence that the Hall technique can significantly reduce major failure rates compared to conventional restorations, as reported by BaniHani et al. (5). Furthermore, a recent SR showed that the Hall Technique is 49% more likely to succeed than conventional treatments and 80% more likely to succeed compared to direct restorations only (57). Its superior performance lies in completely sealing the carious lesion without extensive removal, creating an anaerobic environment that inhibits bacterial progression (84). However, in some contexts, its performance was similar to that of conventional stainless-steel crowns, suggesting that the advantage may depend on lesion type, operator, or clinical setting.

Selective caries excavation was evaluated in nine SRs; four had high confidence. Among these, two reported better performances and two similar results compared to conventional methods. This indicates that selective excavation is a promising, though not universally superior, option. The probable explanation is that it minimizes pulpal risk by leaving affected but firm dentin and reduces operative intervention, improving restoration longevity—particularly important in primary teeth where tooth preservation is prioritized.

For laser use, two SRs were identified; one had high confidence and concluded that laser performance was similar to conventional treatments. This suggests that while technically feasible, laser use has not consistently shown clear superiority—possibly due to factors such as cost, technical complexity, need for specialized equipment, and limited evidence in primary teeth.

Resin infiltration was analyzed in six SRs, four with high confidence, all concluding that resin infiltration performs better than conventional methods. Although most studies have focused on non-cavitated proximal lesions in permanent teeth, these findings suggest a positive transfer to the primary dentition. This could be explained by the technique's early action on porous lesions, inhibiting progression and avoiding the need for more invasive intervention.

Finally, for sealants in carious lesions in primary teeth, four SRs with high confidence reported that one found better performance while the other three found similar results compared to conventional methods. This indicates that sealant use in primary teeth remains a valid option, especially for prevention or management of early lesions, though in deeper cavities it may not outperform other restorative or minimally invasive approaches.

Overall, the apparent benefits of minimally invasive interventions are not uniform across techniques and are sensitive to the methodological quality of the underlying evidence. Consequently, the most reliable inferences in this umbrella review are derived from interventions supported by high-confidence and low-risk systematic reviews, whereas findings based predominantly on low or critically low-confidence reviews should be considered promising but uncertain.

From a comparative clinical perspective, each minimally invasive approach offers distinct advantages depending on lesion characteristics and patient-related factors. Silver diamine fluoride (SDF) presents the strongest evidence for caries arrest, particularly in cavitated lesions and community-based or non-cooperative pediatric settings, due to its high efficacy, low cost, and minimal technical requirements. The Hall technique demonstrates superior survival outcomes in proximal lesions by sealing caries without tissue removal, making it especially suitable for young or anxious children. Resin infiltration is particularly advantageous for non-cavitated proximal lesions, as it arrests lesion progression at an early stage while preserving tooth structure. Atraumatic restorative treatment (ART) is most effective in single-surface cavities and resource-limited environments, offering outcomes comparable to conventional restorations. Selective caries excavation minimizes the risk of pulp exposure while maintaining restoration longevity. In contrast, fluoride varnish and laser-based interventions show more limited or comparable effectiveness and mainly support preventive or adjunctive roles rather than definitive treatment of established lesions.

### 4.2 Implications for clinical practice

The differentiated advantages of each minimally invasive strategy underscore the importance of tailoring clinical decisions to injury severity, child cooperation, and available resources. The findings of this review suggest that minimally invasive strategies play a significant role in managing caries in primary dentition, highlighting the strong support for SDF, whose efficacy has been consistently demonstrated in high-quality reviews, suggesting that clinicians may consider its use in carious primary teeth, particularly when supported by high-confidence evidence and aligned with individual clinical context. Likewise, the Hall technique and resin infiltration show similar levels of benefit, allowing for less invasive treatments, reduced need for anesthesia, and more favorable clinical experiences for children.

Based on the certainty and consistency of the available evidence, minimally invasive interventions can be differentiated according to their level of clinical support. Silver diamine fluoride (SDF), the Hall technique, and resin infiltration are supported by the most robust evidence, as these approaches were consistently evaluated in high-confidence systematic reviews with low risk of bias and demonstrated superior or equivalent outcomes compared with conventional treatments. SDF is particularly supported for caries arrest in cavitated lesions and in non-cooperative children or community-based settings. The Hall technique shows strong support for proximal and multi-surface lesions, with high restoration survival and reduced major failure rates, while resin infiltration is best supported for non-cavitated proximal lesions, where it effectively arrests lesion progression while preserving tooth structure. In contrast, interventions such as atraumatic restorative treatment (ART), selective caries excavation, sealants, fluoride varnish, laser-based therapies, and chemical-mechanical caries removal are supported by more heterogeneous or lower-quality evidence and should therefore be considered context-dependent or adjunctive options rather than universally preferred first-line treatments.

However, variability in the evidence, such as for ART or selective excavation, indicates that not all minimally invasive methods are equally supported. Although the study overlap is low, heterogeneity and bias risk still limit some conclusions. Therefore, professionals should interpret findings cautiously and communicate to parents/guardians that, while the trend favors minimal intervention, not all methods have the same level of evidence.

In resource-limited settings, procedures such as SDF or ART can expand access to treatment, reduce lesion progression, and lower associated costs. In pediatric dental practice, it remains essential to consider each patient's individual context (cooperation level, lesion extent, resource availability) and adopt a personalized approach. Finally, although minimally invasive techniques show great potential and comparable or superior efficacy to conventional treatments, they do not completely replace them, especially in cases of extensive lesions or pulpal involvement.

Importantly, clinical decision-making should explicitly incorporate evidence certainty. Interventions supported by high-confidence systematic reviews may be preferentially considered, whereas techniques supported by heterogeneous or lower-quality evidence should be individualized and discussed with caregivers in terms of potential benefits and uncertainty.

### 4.3 Implications for research

From a research standpoint, this review identifies several relevant gaps. Despite the low overlap, the distribution of confidence across reviews indicates the need for more rigorous studies, particularly for techniques with limited evidence (e.g., laser and chemo-mechanical removal). The existence of nine critically low-confidence reviews reinforces this need.

Moreover, since 25 SRs showed a low risk of bias but 24 had a high risk, future SRs should improve design, reporting, and bias assessment. There is a need for multicenter clinical trials with medium- and long-term follow-up comparing minimally invasive and conventional techniques in primary teeth.

Future studies should also explore patient- and caregiver-centered outcomes (e.g., pain, anxiety, quality of life), not only clinical success or restoration longevity. As noted by BaniHani et al. (5), reports often focus on clinical outcomes without considering the child's experience.

Finally, it is recommended to develop evidence-based clinical guidelines adapted to pediatric dental care settings, to standardize the integration of minimally invasive techniques with conventional management when appropriate. Improving the methodological rigor and transparency of future systematic reviews is essential to strengthen evidence synthesis in pediatric minimal intervention dentistry.

## 5 Conclusions

This umbrella review suggests that minimally invasive interventions can be effective for managing carious lesions in primary teeth; However, the strength of the evidence varies across techniques and depends on the methodological quality of the underlying systematic reviews. When considering findings supported predominantly by high-confidence and low-risk reviews, silver diamine fluoride, the Hall technique, and resin infiltration demonstrate the most consistent benefits for specific lesion types and clinical contexts. For ART, selective caries excavation, and sealants, the evidence is more heterogeneous, supporting their use as context-dependent alternatives rather than universally superior options. Evidence for fluoride varnish, laser-based approaches, and chemo-mechanical caries removal remains limited or methodologically variable, precluding strong conclusions. Overall, these findings support the role of minimally invasive dentistry in pediatric care while emphasizing the need for cautious interpretation and further high-quality research to strengthen clinical recommendations.

## Data Availability

The original contributions presented in the study are included in the article/Supplementary Material, further inquiries can be directed to the corresponding author.
