# Supplementary material for: Effect of minimal intervention on carious lesions in primary teeth. An Umbrella review
Source: Front Dent Med. 2026 Jan 12;6:1751752. doi: 10.3389/fdmed.2025.1751752 (PMC12833399; doi:10.3389/fdmed.2025.1751752)
Supplement: Supplementary file 1 [file Table1.docx]

Supplementary Material 1. Database search strategy

| **Database** | **Search strategy** | **Number of studies** |
| --- | --- | --- |
| Pubmed | ("Tooth, Deciduous"[MeSH Terms] OR "primary dentition"[Title/Abstract] OR "primary teeth"[Title/Abstract] OR "deciduous teeth"[Title/Abstract] OR "temporary teeth"[Title/Abstract] OR "baby teeth"[Title/Abstract] OR "primary tooth"[Title/Abstract] OR "deciduous tooth"[Title/Abstract] OR "temporary tooth"[Title/Abstract] OR "baby tooth"[Title/Abstract]) AND ("Dental Caries"[MeSH Terms] OR "carious lesion*"[Title/Abstract] OR "carious teeth"[Title/Abstract] OR "carious tooth"[Title/Abstract] OR "tooth caries"[Title/Abstract] OR "teeth caries"[Title/Abstract] OR "dental caries"[Title/Abstract] OR "tooth decay"[Title/Abstract] OR "teeth decay"[Title/Abstract] OR "dental decay"[Title/Abstract]) AND ("Dental Atraumatic Restorative Treatment"[MeSH Terms] OR "minimal intervention"[Title/Abstract] OR "minimal* invasive"[Title/Abstract] OR "biological treatment*"[Title/Abstract] OR "biological approach*"[Title/Abstract] OR "ultraconservative"[Title/Abstract] OR "excavation*"[Title/Abstract] OR "caries removal"[Title/Abstract] OR "atraumatic restorative"[Title/Abstract] OR "stepwise removal"[Title/Abstract] OR "silver diamine fluoride"[Title/Abstract] OR "silver diammine fluoride"[Title/Abstract] OR "diammine silver fluoride"[Title/Abstract] OR "diamine silver fluoride"[Title/Abstract] OR "silver fluoride"[Title/Abstract] OR "Hall crown*"[Title/Abstract] OR "Hall technique"[Title/Abstract] OR "preformed metal crown*"[Title/Abstract] OR "sealing"[Title/Abstract] OR " Pit and Fissure Sealants"[MeSH Terms] OR "fissure sealer*"[Title/Abstract] OR "dental sealer*"[Title/Abstract] OR "resin infiltration"[Title/Abstract] OR "ICON system"[Title/Abstract] OR "selective"[Title/Abstract] OR "non-restorative caries"[Title/Abstract] OR "non-operative caries"[Title/Abstract] OR "partial removal"[Title/Abstract] OR "complete removal"[Title/Abstract] OR "incomplete removal"[Title/Abstract] OR "partial excavation"[Title/Abstract] OR "incomplete excavation"[Title/Abstract] OR "indirect pulp"[Title/Abstract] OR "Lasers, Solid-State"[MeSH Terms] OR "Lasers"[MeSH Terms] OR "Laser"[Title/Abstract] OR "Erbium Laser"[Title/Abstract]) AND ("systematic review"[Title/Abstract] OR "meta-analysis"[Title/Abstract]) | 108 |
| Cochrane database | #1 MeSH descriptor: [Tooth, Deciduous] explode all trees  #2 ("primary dentition"):ti,ab,kw OR ("primary teeth"):ti,ab,kw OR ("deciduous teeth"):ti,ab,kw OR ("temporary teeth"):ti,ab,kw OR ("baby teeth"):ti,ab,kw OR ("primary tooth"):ti,ab,kw OR ("deciduous tooth"):ti,ab,kw OR ("temporary tooth"):ti,ab,kw OR ("baby tooth"):ti,ab,kw (Word variations have been searched)  #3 #1 OR #2  #4 MeSH descriptor: [Dental Caries] explode all trees  #5 ("carious lesion"):ti,ab,kw OR ("carious teeth"):ti,ab,kw OR ("carious tooth"):ti,ab,kw OR ("tooth caries"):ti,ab,kw OR ("teeth caries"):ti,ab,kw OR ("dental caries"):ti,ab,kw OR ("tooth decay"):ti,ab,kw OR ("teeth decay"):ti,ab,kw OR ("dental decay"):ti,ab,kw (Word variations have been searched)  #6 #4 OR #5  #7 MeSH descriptor: [Dental Atraumatic Restorative Treatment] explode all trees  #8 MeSH descriptor: [Pit and Fissure Sealants] explode all trees  #9 MeSH descriptor: [Lasers, Solid-State] explode all trees  #10 MeSH descriptor: [Lasers] explode all trees  #11 ("Dental Atraumatic Restorative Treatment"):ti,ab,kw OR ("minimal intervention"):ti,ab,kw OR ("minimal invasive"):ti,ab,kw OR ("biological treatment"):ti,ab,kw OR ("biological approach"):ti,ab,kw OR ("ultraconservative"):ti,ab,kw OR ("excavation"):ti,ab,kw OR ("caries removal"):ti,ab,kw OR ("atraumatic restorative"):ti,ab,kw OR ("stepwise removal"):ti,ab,kw OR ("silver diamine fluoride"):ti,ab,kw OR ("silver diammine fluoride"):ti,ab,kw OR ("diammine silver fluoride"):ti,ab,kw OR ("diamine silver fluoride"):ti,ab,kw OR ("silver fluoride"):ti,ab,kw OR ("Hall crown"):ti,ab,kw OR ("Hall technique"):ti,ab,kw OR ("preformed metal crown"):ti,ab,kw OR ("sealing"):ti,ab,kw OR ("Pit and Fissure Sealants"):ti,ab,kw OR ("fissure sealer"):ti,ab,kw OR ("dental sealer"):ti,ab,kw OR ("resin infiltration"):ti,ab,kw OR ("ICON system"):ti,ab,kw OR ("selective"):ti,ab,kw OR ("non-restorative caries"):ti,ab,kw OR ("non-operative caries"):ti,ab,kw OR ("partial removal"):ti,ab,kw OR ("complete removal"):ti,ab,kw OR ("incomplete removal"):ti,ab,kw OR ("partial excavation"):ti,ab,kw OR ("incomplete excavation"):ti,ab,kw OR ("indirect pulp"):ti,ab,kw OR ("Laser"):ti,ab,kw OR ("Erbium Laser"):ti,ab,kw (Word variations have been searched)  #12 #7 OR #8 OR #9 OR #10 OR #11  #11 #3 AND #6 AND #12 | 22 |
| Scopus | TITLE-ABS-KEY (("primary dentition" OR "primary teeth" OR "deciduous teeth" OR "temporary teeth" OR "baby teeth" OR "primary tooth" OR "deciduous tooth" OR "temporary tooth" OR "baby tooth")) AND TITLE-ABS-KEY (("carious lesion" OR "carious teeth" OR "carious tooth" OR "tooth caries" OR "teeth caries" OR "dental caries" OR "tooth decay" OR "teeth decay" OR "dental decay")) AND TITLE-ABS-KEY (("Dental Atraumatic Restorative Treatment" OR "minimal intervention" OR "minimal invasive" OR "biological treatment" OR "biological approach" OR "ultraconservative" OR "excavation" OR "caries removal" OR "atraumatic restorative" OR "stepwise removal" OR "silver diamine fluoride" OR "silver diammine fluoride" OR "diammine silver fluoride" OR "diamine silver fluoride" OR "silver fluoride" OR "Hall crown" OR "Hall technique" OR "preformed metal crown" OR "sealing" OR " Pit and Fissure Sealants" OR "fissure sealer" OR "dental sealer" OR "resin infiltration" OR "ICON system" OR "selective" OR "non-restorative caries" OR "non-operative caries" OR "partial removal" OR "complete removal" OR "incomplete removal" OR "partial excavation" OR "incomplete excavation" OR "indirect pulp" OR "Lasers" OR "Laser" OR "Erbium Laser")) AND TITLE-ABS-KEY (("systematic review" OR "meta-analysis")) | 141 |
| Web of Science | TS=(("primary dentition" OR "primary teeth" OR "deciduous teeth" OR "temporary teeth" OR "baby teeth" OR "primary tooth" OR "deciduous tooth" OR "temporary tooth" OR "baby tooth")) AND TS=(("carious lesion" OR "carious teeth" OR "carious tooth" OR "tooth caries" OR "teeth caries" OR "dental caries" OR "tooth decay" OR "teeth decay" OR "dental decay")) AND TS=(("Dental Atraumatic Restorative Treatment" OR "minimal intervention" OR "minimal invasive" OR "biological treatment" OR "biological approach" OR "ultraconservative" OR "excavation" OR "caries removal" OR "atraumatic restorative" OR "stepwise removal" OR "silver diamine fluoride" OR "silver diammine fluoride" OR "diammine silver fluoride" OR "diamine silver fluoride" OR "silver fluoride" OR "Hall crown" OR "Hall technique" OR "preformed metal crown" OR "sealing" OR "Pit and Fissure Sealants" OR "fissure sealer" OR "dental sealer" OR "resin infiltration" OR "ICON system" OR "selective" OR "non-restorative caries" OR "non-operative caries" OR "partial removal" OR "complete removal" OR "incomplete removal" OR "partial excavation" OR "incomplete excavation" OR "indirect pulp" OR "Lasers" OR "Laser" OR "Erbium Laser")) AND TS=(("systematic review" OR "meta-analysis")) | 63 |
| Embase | ("primary dentition":ti,ab,kw OR "primary teeth":ti,ab,kw OR "deciduous teeth":ti,ab,kw OR "temporary teeth":ti,ab,kw OR "baby teeth":ti,ab,kw OR "primary tooth":ti,ab,kw OR "deciduous tooth":ti,ab,kw OR "temporary tooth":ti,ab,kw OR "baby tooth":ti,ab,kw) AND ("carious lesion":ti,ab,kw OR "carious teeth":ti,ab,kw OR "carious tooth":ti,ab,kw OR "tooth caries":ti,ab,kw OR "teeth caries":ti,ab,kw OR "dental caries":ti,ab,kw OR "tooth decay":ti,ab,kw OR "teeth decay":ti,ab,kw OR "dental decay":ti,ab,kw) AND ("Dental Atraumatic Restorative Treatment":ti,ab,kw OR "minimal intervention":ti,ab,kw OR "minimal invasive":ti,ab,kw OR "biological treatment":ti,ab,kw OR "biological approach":ti,ab,kw OR "ultraconservative":ti,ab,kw OR "excavation":ti,ab,kw OR "caries removal":ti,ab,kw OR "atraumatic restorative":ti,ab,kw OR "stepwise removal":ti,ab,kw OR "silver diamine fluoride":ti,ab,kw OR "silver diammine fluoride":ti,ab,kw OR "diammine silver fluoride":ti,ab,kw OR "diamine silver fluoride":ti,ab,kw OR "silver fluoride":ti,ab,kw OR "Hall crown":ti,ab,kw OR "Hall technique":ti,ab,kw OR "preformed metal crown":ti,ab,kw OR "sealing":ti,ab,kw OR "Pit and Fissure Sealants":ti,ab,kw OR "fissure sealer":ti,ab,kw OR "dental sealer":ti,ab,kw OR "resin infiltration":ti,ab,kw OR "ICON system":ti,ab,kw OR "selective":ti,ab,kw OR "non-restorative caries":ti,ab,kw OR "non-operative caries":ti,ab,kw OR "partial removal":ti,ab,kw OR "complete removal":ti,ab,kw OR "incomplete removal":ti,ab,kw OR "partial excavation":ti,ab,kw OR "incomplete excavation":ti,ab,kw OR "indirect pulp":ti,ab,kw OR "Lasers":ti,ab,kw OR "Laser":ti,ab,kw OR "Erbium Laser":ti,ab,kw) AND ("systematic review":ti,ab,kw OR "meta-analysis":ti,ab,kw) | 53 |
| Scielo | ("primary dentition" OR "primary teeth" OR "deciduous teeth") AND ("carious lesion" OR "tooth caries" OR "teeth caries" OR "dental caries") AND ("Dental Atraumatic Restorative Treatment" OR "minimal intervention" OR "minimal invasive" OR "atraumatic restorative" OR "silver diamine fluoride" OR "Hall technique" OR "sealing" OR "Pit and Fissure Sealants" OR "fissure sealer" OR "resin infiltration" OR "ICON system" OR "partial removal" OR "indirect pulp" OR "Laser") AND ("systematic review" OR "meta-analysis") | 1 |
| Google Scholar | ("primary dentition" OR "deciduous teeth") + ("tooth caries" OR "teeth caries" OR "dental caries") + ("Dental Atraumatic Restorative Treatment" OR "atraumatic restorative" OR "silver diamine fluoride" OR "Hall technique" OR "sealing" OR "fissure sealer" OR "resin infiltration" OR "indirect pulp" OR "Laser") + ("systematic review" OR "meta-analysis") | 100 |
| Proquest Dissertations and Theses | ("primary dentition" OR "primary teeth" OR "deciduous teeth") AND ("carious lesion" OR "tooth caries" OR "teeth caries" OR "dental caries") AND ("Dental Atraumatic Restorative Treatment" OR "minimal intervention" OR "minimal invasive" OR "atraumatic restorative" OR "silver diamine fluoride" OR "Hall technique" OR "sealing" OR "Pit and Fissure Sealants" OR "fissure sealer" OR "resin infiltration" OR "ICON system" OR "partial removal" OR "indirect pulp" OR "Laser") AND ("systematic review" OR "meta-analysis") NOT ("clinical trial" OR "cross-sectional" OR "review") | 9 |
| OpenGrey | ("primary dentition" OR "primary teeth" OR "deciduous teeth") AND ("carious lesion" OR "tooth caries" OR "teeth caries" OR "dental caries") AND ("Dental Atraumatic Restorative Treatment" OR "minimal intervention" OR "minimal invasive" OR "atraumatic restorative" OR "silver diamine fluoride" OR "Hall technique" OR "sealing" OR "Pit and Fissure Sealants" OR "fissure sealer" OR "resin infiltration" OR "ICON system" OR "partial removal" OR "indirect pulp" OR "Laser") AND ("systematic review" OR "meta-analysis") | 0 |
