# Supplementary material for: Effect of minimal intervention on carious lesions in primary teeth. An Umbrella review
Source: Front Dent Med. 2026 Jan 12;6:1751752. doi: 10.3389/fdmed.2025.1751752 (PMC12833399; doi:10.3389/fdmed.2025.1751752)
Supplement: Supplementary file 2 [file Table2.docx]

Supplementary Material 2. Reason for exclusion of studies

| **Author** | **Reason for exclusion** |
| --- | --- |
| Varughese et al. (1) | Control group with minimally invasive treatment |
| Dadpe et al. (2) |  |
| da Silva et al. (3) |  |
| Mehrotra et al. (4) |  |
| Rogalnikovaitė et al. (5) |  |
| Zaffarano et al. (6) |  |
| Hafiz et al. (7) |  |
| Faria et al. (8) |  |
| Wakhloo et al. (9) |  |
| Doméjean et al. (10) |  |
| Marinho et al. (11) |  |
| Urquhart et al. (12) | Data set of deciduous teeth with permanent |

**References:**

1. Varughese A, Janakiram C, Karuveettil V, James A. Effectiveness of silver diamine fluoride application with atraumatic restorative treatment in arresting the progression of dental caries in children and adults: a systematic review and meta-analysis. *JBI Evid Synth* (2025) 23:1286–1307. doi: 10.11124/JBIES-24-00299

2. Dadpe MV, Shelke PB, Kale YJ, Dahake PT, Kendre SB, Mankar S. Comparative evaluation of Papacarie and Carisolv in effective chemomechanical carious dentin removal in primary teeth: A systematic review. *Dent Res J (Isfahan)* (2025) 22:33. doi: 10.4103/drj.drj_205_23

3. da Silva LB, Magno MB, Fonseca-Gonçalves A, Pintor AVB. ART with or without the aid of chemo-mechanical agents: a systematic review. *Clin Oral Investig* (2024) 28:581. doi: 10.1007/s00784-024-05931-9

4. Mehrotra D, Kodical SR, Naik SS. Comparison of smart burs and chemo-mechanical caries removal systems in primary molars - A systematic review and meta-analysis. *J Indian Soc Pedod Prev Dent* (2024) 42:257–266. doi: 10.4103/jisppd.jisppd_308_24

5. Rogalnikovaitė K, Narbutaitė J, Andruškevičienė V, Bendoraitienė EA, Razmienė J. The Potential of Silver Diamine Fluoride in Non-Operative Management of Dental Caries in Primary Teeth: A Systematic Review. *Medicina (Kaunas)* (2024) 60:1738. doi: 10.3390/medicina60111738

6. Zaffarano L, Salerno C, Campus G, Cirio S, Balian A, Karanxha L, Cagetti MG. Silver Diamine Fluoride (SDF) Efficacy in Arresting Cavitated Caries Lesions in Primary Molars: A Systematic Review and Metanalysis. *Int J Environ Res Public Health* (2022) 19:12917. doi: 10.3390/ijerph191912917

7. Hafiz Z, Allam R, Almazyad B, Bedaiwi A, Alotaibi A, Almubrad A. Effectiveness of Silver Diamine Fluoride in Arresting Caries in Primary and Early Mixed Dentition: A Systematic Review. *Children (Basel)* (2022) 9:1289. doi: 10.3390/children9091289

8. Faria LV, Fernandes T de O, Guimarães LS, Cajazeira MRR, Antunes LS, Antunes LAA. Does selective caries removal in combination with antimicrobial photodynamic therapy affect the clinical performance of adhesive restorations of primary or permanent teeth? A systematic review with meta-analysis. *J Clin Pediatr Dent* (2022) 46:1–14. doi: 10.22514/jocpd.2022.002

9. Wakhloo T, Reddy SG, Sharma SK, Chug A, Dixit A, Thakur K. Silver Diamine Fluoride Versus Atraumatic Restorative Treatment in Pediatric Dental Caries Management: A Systematic Review and Meta-analysis. *J Int Soc Prev Community Dent* (2021) 11:367–375. doi: 10.4103/jispcd.JISPCD_83_21

10. Doméjean S, Ducamp R, Léger S, Holmgren C. Resin infiltration of non-cavitated caries lesions: A systematic review. *Medical Principles and Practice* (2015) 24:216–221. doi: 10.1159/000371709

11. Marinho V, Higgins J, Sheiham A, Logan S. One topical fluoride (toothpastes, or mouthrinses, or gels, or varnishes) versus another for preventing dental caries in children and adolescents. *Cochrane Database Syst Rev* (2004) doi: 10.1002/14651858.CD002780.pub2

12. Urquhart O, Tampi MP, Pilcher L, Slayton RL, Araujo MWB, Fontana M, Guzmán-Armstrong S, Nascimento MM, Nový BB, Tinanoff N, et al. Nonrestorative Treatments for Caries: Systematic Review and Network Meta-analysis. *J Dent Res* (2019) 98:14–26. doi: 10.1177/0022034518800014
