# Supplementary material for: Effect of minimal intervention on carious lesions in primary teeth. An Umbrella review
Source: Front Dent Med. 2026 Jan 12;6:1751752. doi: 10.3389/fdmed.2025.1751752 (PMC12833399; doi:10.3389/fdmed.2025.1751752)
Supplement: Supplementary file 3 [file Table3.docx]

Supplementary Material 3. Characteristics of included studies

| **Authors** | **Year** | **Study design** | **Country** | **Included study design** | **Number of studies (qualitative / quantitative)** | **Register** | **PRISMA** | **GRADE** | **ROBIS** | **Outcomes** | | | **Conclusions** |
| --- | --- | --- | --- | --- | --- | --- | --- | --- | --- | --- | --- | --- | --- |
| Inchingolo et al. (1) | 2025 | SR | Italy | RCT, C, CS | 12/0 | Yes | Yes | No | Low | Overall results indicate that the HT is comparable or superior to CTs in terms of clinical success. | | The HT represents a promising, biologically oriented option for the management of carious lesions in deciduous molars. | |
| Mohapatra et al. (2) | 2025 | SR | India | RCT | 4/0 | Yes | Yes | Yes | Low | No difference was found in the clinical performance of SMART and conventional drill and fill. | | SMART technique have comparable clinical performance to the conventional drill and fill for carious primary teeth of children. SMART can be used to treat asymptomatic deep carious lesions as well as apprehensive children. | |
| Dipalma et al. (3) | 2025 | SR | Italy | RCT | 18/0 | Yes | Yes | No | High | ART effectively managed dental caries, especially with high-viscosity GIC. | | ART is a reliable, minimally invasive technique for pediatric restorative dentistry. | |
| Tasleem et al. (4) | 2025 | SR and MA | Saudi Arabia and Pakistan | RCT | 13/13 | Yes | Yes | Yes | Low | General | OR = 0.31 (0.18 – 0.51) | Microinvasive techniques are superior to noninvasive interventions for the treatment of non-cavitated proximal lesions in primary dentition. | |
| Bukhari et al. (5) | 2025 | SR and MA | Saudi Arabia | RCT | 37/34 | Yes | No | No | High | General | MD = -0.27 (-0.46 – -0.07) | There is a benefit of SDF application compared to no treatment or placebo in caries arrest and prevention of new caries in primary dentition with follow-up intervals ranging from 3-36 months. | |
|  |  |  |  |  |  |  |  |  |  |  | OR = 4.12 (3.12 – 5.44) |  |  |
| Muntean et al. (6) | 2024 | SR | Romania, Italy and United Kingdom | RCT | 21/0 | Yes | Yes | No | Low | SDF is effective non-invasive way to prevent and arrest caries in temporary teeth. | | SDF is a practical, accessible and effective non-invasive way to prevent and arrest caries in temporary teeth. | |
| Vishwanathaiah et al. (7) | 2024 | SR | Saudi Arabia, India and United State | RCT, NRCT | 15/0 | Yes | Yes | No | Low | Most studies in this review consistently suggested that SDF is effective in arresting caries. | | The evidence suggests that SDF is effective in arresting dental caries in children. | |
| Alqalaleef et al. (8) | 2024 | SR and MA | Saudi Arabia | RCT | 20/12 | Yes | Yes | Yes | Low | General | OR = 18.10 (3.89 – 84.15) | SDF could be an invaluable tool in combating dental caries, providing a less invasive and potentially more cost-effective alternative to traditional treatment methods. | |
| Inchingolo et al. (9) | 2024 | SR | Italy and United Kingdom | RCT, RS, NS, PCS | 13/0 | Yes | Yes | No | Low | The studies reviewed consistently support the effectiveness of SDF in stopping the progression of existing caries in deciduous molars. | | SDF could be an invaluable tool in combating dental caries, providing a less invasive and potentially more cost-effective alternative to traditional treatment methods. | |
| Chua et al. (10) | 2023 | SR and MA | Singapore and Qatar | CT, C | 5/4 | Yes | Yes | No | Low | General | OR = 1.06 (0.65 – 1.73) | Preformed metal crowns placed using either the HT or CT confers similar overall success and survival rates. Given the favourable outcomes, greater consideration may be given towards using the HT as part of standard treatment procedures in carious primary molars in children. | |
| Cebula et al. (11) | 2023 | SR and MA | Germany | RCT | 11/11 | Yes | Yes | Yes | Low | General | OR = 0.41 (0.29 – 0.57) | There is firm evidence for resin infiltration arresting proximal caries lesions in primary teeth. | |
| Ramamurthy et al. (12) | 2022 | SR and MA | Malaysia, United Kingdom and Chile | RCT | 9/5 | Yes | Yes | Yes | Low | General | OR = 0.76 (0.41 – 1.42) | The effectiveness of pit and fissure sealants and the relative effectiveness of different types of sealants in preventing caries on the occlusal surfaces of primary teeth has yet to be established. | |
| Tedesco et al. (13) | 2022 | SR and MA | Brazil, United Kingdom and United State | RCT | 11/11 | Yes | Yes | Yes | Low | General - Proximal - Resin infiltration | OR = 6.9 (2.00 – 24.00) | The limited number of included studies, most with a high risk of bias and lack of hard outcomes, such as frank cavitation, makes it not feasible to recommend a specific management approach for initial caries lesion control in primary teeth with a high certainty of evidence | |
|  |  |  |  |  |  |  |  |  |  | General - Proximal - Fluoride varnish | OR = 2.1 (0.52 – 9.00) |  |  |
|  |  |  |  |  |  |  |  |  |  | General - Occlusal - Fluoride varnish | OR = 1.1 (0.19 – 6.0) |  |  |
|  |  |  |  |  |  |  |  |  |  | General - buccal/lingual - Fluoride varnish | OR = 0.99 (0.10 – 10.0) |  |  |
| Chaudhari et al. (14) | 2022 | SR | India | RCT | 6/0 | Yes | Yes | No | High | The survival rate of single surface and multiple surface in primary dentition treated according to the ART compared with conventional treatment was found to be similar. | | The ART approach is equally helpful in managing dental caries in children and this method may be considered a useful intervention in clinical practice to enhance the dental health of children. | |
| Hu et al. (15) | 2022 | SR and MA | Singapore, United Kingdom and Germany | RCT, CCT | 8/5 | Yes | Yes | No | Low | General | RR = 1.49 (1.15 – 1.93) | HT is successful option for the management of caries in primary teeth, particularly for proximal or multisurface dentine lesions. | |
| Schwendicke et al. (16) | 2021 | SR and MA | Germany, United Kingdom, Denmark and United State | RCT | 27/23 | Yes | Yes | Yes | Low | General - Sealant | OR = 5.00 (0.51 – 49.27) | Compared with conventional restoration, there were lower numbers of failures with Hall technique and selective excavation in the primary dentition. | |
|  |  |  |  |  |  |  |  |  |  | General - Hall | OR = 8.35 (3.73 – 18.68) |  |  |
|  |  |  |  |  |  |  |  |  |  | General - Selective excavation | OR = 4.43 (1.04 – 18.77) |  |  |
| Chen et al. (17) | 2021 | SR and MA | China | RCT | 22/22 | No | Yes | Yes | High | General - Resin infiltration | OR = 0.21 (0.15 – 0.30) | Resin infiltration and sealing with sealant were more efficacious than non-invasive treatments for halting non-cavitated proximal lesions. | |
|  |  |  |  |  |  |  |  |  |  | General - Sealant | OR = 0.23 (0.18 – 0.30) |  |  |
| Santamaría et al. (18) | 2020 | SR | Germany and United State | RCT | 6/0 | No | Yes | No | Low | For treatments involving no carious tissue removal, the HT showed lower treatment failure for approximal carious lessions compared to complete caries removal and filling. For the treatment of deep carious lesions, techniques involving selective caries removal showed a reduction in the incidence of pulp exposure. | | Less invasive caries approaches involving selective caries removal or HT seem advantageous in comparison to complete caries removal for patients presenting with vital, symptomless, carious dentin lesions in primary teeth. | |
| Pagano et al. (19) | 2020 | SR | Italy | RCT, CCT | 10/0 | No | Yes | No | High | General - Laser | RR = 0.30 (0.11 – 0.78) | Lasers were found to be effectiveness in preventing caries in primary teeth. | |
|  |  |  |  |  |  |  |  |  |  | General - Sealant | RR = 0.67 (0.35 – 1.26) |  |  |
| Aïem et al. (20) | 2020 | SR and MA | France | RCT | 10/8 | No | Yes | No | High | General - Selective excavation | OR = 0.10 (0.04 – 0.25) | Selective caries removal may result in lower pulp exposure risk than complete caries removal. | |
| Jabin et al. (21) | 2020 | SR | India | CCT | 4/0 | No | Yes | No | High | The cumulative results of the studies showed that 38% SDF application is efficacious and safe for the control of dental caries in primary teeth | | 38% SDF is one of the best treatment approaches in control of dental caries in primary dentition. | |
| Oliveira et al. (22) | 2019 | SR and MA | Brazil and United State | CCT | 4/2 | Yes | Yes | No | High | General | WMD = -1.15 (-1.48 – 0.82) | When applied to caries lesions in primary teeth, SDF compared to no treatment, placebo or fluoride varnish appears to effectively prevent dental caries in the entire dentition. | |
| Elrashid et al. (23) | 2019 | SR and MA | Saudi Arabia | RCT | 7/5 | No | Yes | Yes | High | General | RR = 0.48 (0.30 – 0.75) | The available evidence conveys high confidence that proximal resin infiltration has superior efficacy in slowing/arresting the carious lesions’ progression rate in comparison to conventional management modalities. | |
| Trieu et al. (24) | 2019 | SR and MA | United State | RCT | 6/6 | No | Yes | No | High | General | OR = 2.99 (2.03 – 4.42) | SDF is a effective dentine caries arresting agent in primary teeth | |
| Pedrotti et al. (25) | 2019 | SR and MA | Brazil | RCT | 4/4 | No | Yes | Yes | High | General | OR = 1.74 (1.01 – 3.00) | Selective carious tissue removal of soft dentin may increase the risk of experiencing restoration failure in primary teeth | |
| Li et al. (26) | 2018 | SR and MA | China | RCT | 7/7 | No | Yes | No | High | General | OR = 1.40 (0.69 – 2.84) | The efficacy of selective caries removal appears comparable to that of non-selective caries removal in children. | |
| Deng et al. (27) | 2018 | SR and MA | China | RCT, CCT | 15/10 | No | Yes | No | High | General | MD = 0.57 (0.04 – 1.09) | Papacarie exerts a positive effect in reducing the bacteria and decreases the pain during caries removal in primary teeth. | |
| Tedesco et al. (28) | 2018 | SR and MA | Brazil and Switzerland | RCT | 15/13 | Yes | Yes | Yes | Low | General - Occlusal - ART | RR = 1.01 (0.99 – 1.03) | The treatment of dentin caries lesions in primary teeth depends on the progression depth and surface involved. However, few studies exist, and most have a high risk of bias to provide enough evidence to strongly recommend the best treatment option. | |
|  |  |  |  |  |  |  |  |  |  | General - Occlusal - SDF | RR = 1.11 (1.03 – 1.20) |  |  |
|  |  |  |  |  |  |  |  |  |  | General - Occlusoproximal - ART | RR = 0.98 (0.94 – 1.04) |  |  |
|  |  |  |  |  |  |  |  |  |  | General - Occlusoproximal - Hall | RR = 1.30 (1.09 – 1.54) |  |  |
| Ruengrungsom et al. (29) | 2018 | SR | Australia and China | RCT, RS, LUS | 65/0 | No | Yes | No | High | The GIC-ART technique is an alternative option for single-surface (occlusal) restorations in primary teeth. However, the application of the GIC-ART technique for load-bearing approximal restorations should be carefully considered before employing this option, especially in primary teeth. | | The GIC-ART technique is an alternative option for single-surface (occlusal) restorations in primary teeth. | |
| Dorri et al. (30) | 2017 | SR and MA | United Kingdom, Spain and Chile | RCT | 15/13 | Yes | Yes | Yes | Low | General | OR = 1.60 (1.13 – 2.27) | Low-quality evidence suggests that ART using H-GIC may have a higher risk of restoration failure than conventional treatment for caries lesions in primary teeth. | |
| Contreras et al. (31) | 2017 | SR | Puerto Rico | RCT | 7/0 | No | Yes | No | High | The literature indicates that SDF is a preventive treatment for dental caries in community settings. At concentrations of 30% and 38%, SDF shows potential as an alternative treatment for caries arrest in the primary dentition. | | SDF, at concentrations of 30% and 38%, is more effective than other preventive management strategies for arresting dentinal caries and show potential as a caries preventive treatment in the primary dentition. | |
| Papageorgiou et al. (32) | 2017 | SR and MA | Switzerland, Greece and Austria | RCT | 16/16 | Yes | Yes | Yes | Low | General | RR = 1.06 (0.45 – 2.49) | The performance of pit and fissure sealants does not seem to be negatively affected by mouth side, jaw, and tooth type. | |
| Chibinski et al. (33) | 2017 | SR and MA | Brazil | RCT | 11/4 | Yes | Yes | Yes | Low | General | RR = 2.54 (1.67 – 3.85) | SDF is more effective than other active treatments or placebo for caries arrestment in primary teeth. | |
| Tedesco et al. (34) | 2017 | SR and MA | Brazil and Netherlands | RCT | 4/4 | Yes | Yes | No | Low | General | OR = 0.89 (0.57 – 1.37) | ART restorations have similar survival rates compared to conventional treatment and are a viable option to restore occlusoproximal cavities in primary molars. | |
| Montedori et al. (35) | 2016 | SR and MA | Italy | RCT | 9/9 | Yes | Yes | Yes | Low | General | RR = 1.00 (0.99 – 1.01) | Evidence was insufficient to support the use of laser as an alternative to traditional drill therapy for caries removal. | |
| Duangthip et al. (36) | 2016 | SR | China | RCT, CCT | 9/0 | No | No | No | High | The use of minimally invasive approaches has demonstrated clinical advantages in caries removal and cavity preparation. High caries arrest rates have been achieved through the application of silver diamine fluoride. | | Minimally invasive approaches are advantageous in operative caries management in primary teeth in preschool children. | |
| Gao et al. (37) | 2016 | SR and MA | China and Japan | CT | 19/8 | No | Yes | No | High | General | OR = 0.81 (0.68 – 0.89) | 38% SDF can effectively arrest caries among children. | |
| Innes et al. (38) | 2015 | SR and MA | United Kingdom and Germany | RCT | 5/3 | Yes | No | Yes | Low | General | RR = 0.18 (0.06 – 0.56) | Crowns fitted using the HT may reduce discomfort at the time of treatment compared to fillings. | |
| Duangthip et al. (39) | 2015 | SR | China | RCT, LS | 4/0 | No | No | No | High | The topical applications of SDF solution could arrest dentin caries in preschool children. | | There is limited evidence to support the effectiveness of SDF applications in arresting or slowing down the progression of active dentin caries in primary teeth in preschool children. | |
| Dorri et al. (40) | 2015 | SR and MA | United Kingdom and Germany | RCT | 8/8 | Yes | Yes | Yes | Low | General - Proximal - Sealant | OR = 0.26 (0.13 – 0.53) | The available evidence shows that micro-invasive treatment of proximal caries lesions arrests non-cavitated enamel and initial dentinal lesions (limited to outer third of dentine, based on radiograph) and is significantly more eKective than non-invasive professional treatment (e.g. fluoride varnish) or advice (e.g. to floss). | |
|  |  |  |  |  |  |  |  |  |  | General - Proximal - Resin infiltration | OR = 0.15 (0.06 – 0.39) |  |  |
| Lai et al. (41) | 2015 | SR and MA | Italy and Sweden | RCT, CCT | 10/7 | No | Yes | No | High | General | OR = 0.33 (0.01 – 8.22) | The clinical efficacy of chemo-mechanical removal with Carisolv seems as reliable as the rotary instruments. | |
| Schwendicke et al. (42) | 2013 | SR and MA | Germany | RCT | 10/10 | No | No | Yes | High | General | OR = 0.97 (0.64 – 1.46) | Incomplete caries removal seems advantageous compared with complete excavation, especially in proximity to the pulp. | |
| Ricketts et al. (43) | 2013 | SR and MA | United Kingdom | RCT | 8/8 | Yes | Yes | Yes | Low | General | RR = 0.33 (0.01 – 7.62) | When partial caries removal was carried there was insufficient evidence to determine whether or not there is a difference in risk of restoration failure compared to complete caries removal. | |
| Raggio et al. (44) | 2013 | SR and MA | Brazil | RCT | 3/3 | No | No | No | High | General | OR = 1.18 (0.93 – 1.49) | Atraumatic restorative treatment restorations performed with H- GIC present similar survival/success rates to conventional approach using composite resin or amalgam for occlusoproximal restorations in primary teeth. | |
| Marinho et al. (45) | 2013 | SR and MA | United Kingdom | RCT, CCT | 22/21 | Yes | Yes | Yes | Low | General | RR = 0.81 (0.62 – 1.06) | The review suggests a substantial caries inhibiting effect of fluoride varnish in primary teeth. | |
| Ferreira et al. (46) | 2012 | SR | Brazil | RCT | 3/0 | No | No | No | High | Partial removal of carious tissue favors the arrest of dental caries lesions. | | Partial removal of carious tissue favors the arrest of dental caries lesions. | |
| de Amorim et al. (47) | 2012 | SR and MA | Brazil and Netherlands | OS | 27/27 | No | No | No | High | The survival rates of single-surface and multiple-surface ART restorations in primary teeth over the first 2 years were 93% (CI, 91–94%) and 62% (CI, 51–73%), respectively. The mean annual dentine lesion incidence rate, in pits and fissures previously sealed using ART, over the first 3 years was 1%. | | ART can safely be used in single surface cavities in primary teeth. | |
| Ricketts et al. (48) | 2006 | SR | United Kingdom | RCT | 4/0 | Yes | Yes | No | Low | Partial caries removal in symptomless, primary or permanent teeth reduces the risk of pulp exposure. We found no detriment to the patient in terms of pulpal symptoms in this procedure and no reported premature loss or deterioration of the restoration. | | There is no difference in progression of decay and longevity of restorations irrespective of whether the removal of decay had been minimal (ultraconservative) or complete. | |
| van 't Hof et al. (49) | 2006 | SR and MA | Netherlands | OS | 28/28 | No | No | No | High | High mean survival rates for single-surface ART restorations using H-GIC in primary dentitions over 3 years were found (95% after 1 year to 86% after 3 years). The mean annual failure rates for single-surface and for multiple-surface ART restorations using high-viscosity glass-ionomer in primary dentitions in primary dentitions are 4.7% and 17%, respectively. | | The single-surface ART restorations using H-GIC in both primary and permanent dentitions show high survival rates. | |

SR = Systematic review; MA = Meta-analysis; RCT = Randomized clinical trial; C = Cohort study; CS = Cross-sectional study; NRCT = Non-randomized clinical trial; RS = Retrospective study; NS = Naturalistic study; PCS = Prospective clinical study; CT = Clinical trial; CCT = Controlled clinical trial; LUS = Longitudinal uncontrolled (single-arm) studies; LS = Longitudinal study; OS = Observational study; HT = Hall technique; CT = Conventional technique; ART = Atraumatic restorative treatment; SMART = Silver-modified atraumatic restorative therapy; SDF = Silver diamine fluoride; GIC = Glass ionomer cement; H-GIC = High-viscous glass ionomer cement; OR = Odds ratio; RR = Risk ratio; MD = Mean difference; WMD = Weighted mean difference

**References**

1. Inchingolo AM, Inchingolo AD, Morolla R, Riccaldo L, Guglielmo M, Palumbo I, Palermo A, Francesco F, Dipalma G. Pre-formed crowns and pediatric dentistry: a systematic review of different techniques of restorations. *J Clin Pediatr Dent* (2025) 49:1–13. doi: 10.22514/jocpd.2025.001

2. Mohapatra S, Mohandas R. Clinical Outcome Success of Silver-Modified Atraumatic Restorative Treatment (SMART) in Treating Children with Dental Caries in Primary Teeth: A Systematic Review. *JHASNU* (2025) 15:4–10. doi: 10.1055/s-0044-1788659

3. Dipalma G, Inchingolo AM, Casamassima L, Nardelli P, Ciccarese D, De Sena P, Inchingolo F, Palermo A, Severino M, Maspero CMN, et al. Effectiveness of Dental Restorative Materials in the Atraumatic Treatment of Carious Primary Teeth in Pediatric Dentistry: A Systematic Review. *Children (Basel)* (2025) 12:511. doi: 10.3390/children12040511

4. Tasleem R, Alqahtani SA, Abogazalah N, Almubarak H, Riaz A, Ali SS, Allana Z. Microinvasive interventions in the management of proximal caries lesions in primary and permanent teeth- systematic review and meta-analysis. *BMC Oral Health* (2025) 25:48. doi: 10.1186/s12903-024-05400-5

5. Bukhari OM. Effectiveness of topical silver diamine fluoride for management of dental caries in children and early adolescents: A systematic review and meta-analysis. *Rom J Oral Rehabil* (2025) 17:975–992. doi: 10.62610/RJOR.2025.2.17.89

6. Muntean A, Mzoughi SM, Pacurar M, Candrea S, Inchingolo AD, Inchingolo AM, Ferrante L, Dipalma G, Inchingolo F, Palermo A, et al. Silver Diamine Fluoride in Pediatric Dentistry: Effectiveness in Preventing and Arresting Dental Caries-A Systematic Review. *Children (Basel)* (2024) 11:499. doi: 10.3390/children11040499

7. Vishwanathaiah S, Maganur PC, Syed AA, Kakti A, Hussain Jaafari AH, Albar DH, Renugalakshmi A, Jeevanandan G, Khurshid Z, Ali Baeshen H, et al. Effectiveness of silver diamine fluoride (SDF) in arresting coronal dental caries in children and adolescents: a systematic review. *J Clin Pediatr Dent* (2024) 48:27–40. doi: 10.22514/jocpd.2024.101

8. Alqalaleef SS, Alnakhli RA, Ezzat Y, AlQadi HI, Aljilani AD, Natto ZS. The role of silver diamine fluoride as dental caries preventive and arresting agent: a systematic review and meta-analysis. *Front Oral Health* (2024) 5:1492762. doi: 10.3389/froh.2024.1492762

9. Inchingolo F, Inchingolo AD, Latini G, Sardano R, Riccaldo L, Mancini A, Palermo A, Inchingolo AM, Dipalma G. Caries in primary molars: is silver diamine fluoride effective in prevention and treatment? A systematic review. *Appl Sci* (2024) 14:2055. doi: 10.3390/app14052055

10. Chua DR, Tan BL, Nazzal H, Srinivasan N, Duggal MS, Tong HJ. Outcomes of preformed metal crowns placed with the conventional and Hall techniques: A systematic review and meta-analysis. *Int J Paediatr Dent* (2023) 33:141–157. doi: 10.1111/ipd.13029

11. Cebula M, Göstemeyer G, Krois J, Pitchika V, Paris S, Schwendicke F, Effenberger S. Resin Infiltration of Non-Cavitated Proximal Caries Lesions in Primary and Permanent Teeth: A Systematic Review and Scenario Analysis of Randomized Controlled Trials. *J Clin Med* (2023) 12:727. doi: 10.3390/jcm12020727

12. Ramamurthy P, Rath A, Sidhu P, Fernandes B, Nettem S, Fee PA, Zaror C, Tanya C. Walsh T. Sealants for preventing dental caries in primary teeth. *Cochrane Database Syst Rev* (2022) 2022: doi: 10.1002/14651858.CD012981.pub2

13. Tedesco TK, Calvo AFB, Pássaro AL, Araujo MP, Ladewig NM, Scarpini S, Lara JS, Braga MM, Gimenez T, Raggio DP. Nonrestorative treatment of initial caries lesion in primary teeth: a systematic review and network meta-analysis. *Acta Odontol Scand* (2022) 80:1–8. doi: 10.1080/00016357.2021.1928748

14. Chaudhari HG, Patil RU, Jathar PN, Jain CA. A systematic review of randomized controlled trials on survival rate of atraumatic restorative treatment compared with conventional treatment on primary dentition. *J Indian Soc Pedod Prev Dent* (2022) 40:112–117. doi: 10.4103/jisppd.jisppd_119_22

15. Hu S, BaniHani A, Nevitt S, Maden M, Santamaria RM, Albadri S. Hall technique for primary teeth: A systematic review and meta-analysis. *Jpn Dent Sci Rev* (2022) 58:286–297. doi: 10.1016/j.jdsr.2022.09.003

16. Schwendicke F, Walsh T, Lamont T, Al-Yaseen W, Bjørndal L, Clarkson JE, Fontana M, Gomez Rossi J, Göstemeyer G, Levey C, et al. Interventions for treating cavitated or dentine carious lesions. *Cochrane Database Syst Rev* (2021) 7:CD013039. doi: 10.1002/14651858.CD013039.pub2

17. Chen Y, Chen D, Lin H. Infiltration and sealing for managing non-cavitated proximal lesions: a systematic review and meta-analysis. *BMC Oral Health* (2021) 21:13. doi: 10.1186/s12903-020-01364-4

18. Santamaría RM, Abudrya MH, Gul G, Mourad MS, Felix Gomez GF, Ferreira Zandona AGF. How to Intervene in the Caries Process: Dentin Caries in Primary Teeth. *Caries Res* (2020) 54:306–323. doi: 10.1159/000508899

19. Pagano S, Lombardo G, Orso M, Abraha I, Capobianco B, Cianetti S. Lasers to prevent dental caries: a systematic review. *BMJ Open* (2020) 10:e038638. doi: 10.1136/bmjopen-2020-038638

20. Aïem E, Joseph C, Garcia A, Smaïl-Faugeron V, Muller-Bolla M. Caries removal strategies for deep carious lesions in primary teeth: Systematic review. *Int J Paediatr Dent* (2020) 30:392–404. doi: 10.1111/ipd.12616

21. Jabin Z, Vishnupriya V, Agarwal N, Nasim I, Jain M, Sharma A. Effect of 38% silver diamine fluoride on control of dental caries in primary dentition: A Systematic review. *J Family Med Prim Care* (2020) 9:1302–1307. doi: 10.4103/jfmpc.jfmpc_1017_19

22. Oliveira BH, Rajendra A, Veitz-Keenan A, Niederman R. The Effect of Silver Diamine Fluoride in Preventing Caries in the Primary Dentition: A Systematic Review and Meta-Analysis. *Caries Res* (2019) 53:24–32. doi: 10.1159/000488686

23. Elrashid AH, Alshaiji BS, Saleh SA, Zada KA, Baseer MA. Efficacy of Resin Infiltrate in Noncavitated Proximal Carious Lesions: A Systematic Review and Meta-Analysis. *J Int Soc Prev Community Dent* (2019) 9:211–218. doi: 10.4103/jispcd.JISPCD_26_19

24. Trieu A, Mohamed A, Lynch E. Silver diamine fluoride versus sodium fluoride for arresting dentine caries in children: a systematic review and meta-analysis. *Sci Rep* (2019) 9:2115. doi: 10.1038/s41598-019-38569-9

25. Pedrotti D, Cavalheiro CP, Casagrande L, de Araújo FB, Pettorossi Imparato JC, de Oliveira Rocha R, Lenzi TL. Does selective carious tissue removal of soft dentin increase the restorative failure risk in primary teeth?: Systematic review and meta-analysis. *J Am Dent Assoc* (2019) 150:582-590.e1. doi: 10.1016/j.adaj.2019.02.018

26. Li T, Zhai X, Song F, Zhu H. Selective versus non-selective removal for dental caries: a systematic review and meta-analysis. *Acta Odontol Scand* (2018) 76:135–140. doi: 10.1080/00016357.2017.1392602

27. Deng Y, Feng G, Hu B, Kuang Y, Song J. Effects of Papacarie on children with dental caries in primary teeth: a systematic review and meta-analysis. *Int J Paediatr Dent* (2018) 28:361–372. doi: 10.1111/ipd.12364

28. Tedesco TK, Gimenez T, Floriano I, Montagner AF, Camargo LB, Calvo AFB, Morimoto S, Raggio DP. Scientific evidence for the management of dentin caries lesions in pediatric dentistry: A systematic review and network meta-analysis. *PLoS One* (2018) 13:e0206296. doi: 10.1371/journal.pone.0206296

29. Ruengrungsom C, Palamara JEA, Burrow MF. Comparison of ART and conventional techniques on clinical performance of glass-ionomer cement restorations in load bearing areas of permanent and primary dentitions: A systematic review. *J Dent* (2018) 78:1–21. doi: 10.1016/j.jdent.2018.07.008

30. Dorri M, Martinez-Zapata MJ, Walsh T, Marinho VC, Sheiham Deceased A, Zaror C. Atraumatic restorative treatment versus conventional restorative treatment for managing dental caries. *Cochrane Database Syst Rev* (2017) 12:CD008072. doi: 10.1002/14651858.CD008072.pub2

31. Contreras V, Toro MJ, Elías-Boneta AR, Encarnación-Burgos A. Effectiveness of silver diamine fluoride in caries prevention and arrest: a systematic literature review. *Gen Dent* (2017) 65:22–29.

32. Papageorgiou SN, Dimitraki D, Kotsanos N, Bekes K, van Waes H. Performance of pit and fissure sealants according to tooth characteristics: A systematic review and meta-analysis. *J Dent* (2017) 66:8–17. doi: 10.1016/j.jdent.2017.08.004

33. Chibinski AC, Wambier LM, Feltrin J, Loguercio AD, Wambier DS, Reis A. Silver Diamine Fluoride Has Efficacy in Controlling Caries Progression in Primary Teeth: A Systematic Review and Meta-Analysis. *Caries Res* (2017) 51:527–541. doi: 10.1159/000478668

34. Tedesco TK, Calvo AFB, Lenzi TL, Hesse D, Guglielmi CAB, Camargo LB, Gimenez T, Braga MM, Raggio DP. ART is an alternative for restoring occlusoproximal cavities in primary teeth - evidence from an updated systematic review and meta-analysis. *Int J Paediatr Dent* (2017) 27:201–209. doi: 10.1111/ipd.12252

35. Montedori A, Abraha I, Orso M, D’Errico PG, Pagano S, Lombardo G. Lasers for caries removal in deciduous and permanent teeth. *Cochrane Database Syst Rev* (2016) 2016: doi: 10.1002/14651858.CD010229.pub2

36. Duangthip D, Jiang M, Chu CH, Lo ECM. Restorative approaches to treat dentin caries in preschool children: systematic review. *Eur J Paediatr Dent* (2016) 17:113–121.

37. Gao SS, Zhao IS, Hiraishi N, Duangthip D, Mei ML, Lo ECM, Chu CH. Clinical Trials of Silver Diamine Fluoride in Arresting Caries among Children: A Systematic Review. *JDR Clin Trans Res* (2016) 1:201–210. doi: 10.1177/2380084416661474

38. Innes NPT, Ricketts D, Chong LY, Keightley AJ, Lamont T, Santamaría RM. Preformed crowns for decayed primary molar teeth. *Cochrane Database Syst Rev* (2015) 2015:CD005512. doi: 10.1002/14651858.CD005512.pub3

39. Duangthip D, Jiang M, Chu CH, Lo ECM. Non-surgical treatment of dentin caries in preschool children--systematic review. *BMC Oral Health* (2015) 15:44. doi: 10.1186/s12903-015-0033-7

40. Dorri M, Dunne SM, Walsh T, Schwendicke F. Micro-invasive interventions for managing proximal dental decay in primary and permanent teeth. *Cochrane Database Syst Rev* (2015) 2015:CD010431. doi: 10.1002/14651858.CD010431.pub2

41. Lai G, Lara Capi C, Cocco F, Cagetti MG, Lingström P, Almhöjd U, Campus G. Comparison of Carisolv system vs traditional rotating instruments for caries removal in the primary dentition: A systematic review and meta-analysis. *Acta Odontol Scand* (2015) 73:569–580. doi: 10.3109/00016357.2015.1023353

42. Schwendicke F, Dörfer C, Paris S. Incomplete Caries Removal: A Systematic Review and Meta-analysis. *J Dent Res* (2013) 92:306–314. doi: 10.1177/0022034513477425

43. Ricketts D, Lamont T, Innes NPT, Kidd E, Clarkson JE. Operative caries management in adults and children. *Cochrane Database Syst Rev* (2013) 28:CD003808. doi: 10.1002/14651858.CD003808.pub3

44. Raggio DP, Hesse D, Lenzi TL, Guglielmi CAB, Braga MM. Is Atraumatic restorative treatment an option for restoring occlusoproximal caries lesions in primary teeth? A systematic review and meta-analysis. *Int J Paediatr Dent* (2013) 23:435–443. doi: 10.1111/ipd.12013

45. Marinho V, Worthington H, Walsh T, Clarkson J. Fluoride varnishes for preventing dental caries in children and adolescents. *Cochrane Database Syst Rev* (2013) 2013:CD002279. doi: 10.1002/14651858.CD002279.pub2

46. Ferreira JMS, Pinheiro SL, Sampaio FC, de Menezes VA. Caries removal in primary teeth--a systematic review. *Quintessence Int* (2012) 43:e9-15.

47. de Amorim RG, Leal SC, Frencken JE. Survival of atraumatic restorative treatment (ART) sealants and restorations: a meta-analysis. *Clin Oral Investig* (2012) 16:429–441. doi: 10.1007/s00784-011-0513-3

48. Ricketts DNJ, Kidd E a. M, Innes N, Clarkson J. Complete or ultraconservative removal of decayed tissue in unfilled teeth. *Cochrane Database Syst Rev* (2006) 19:CD003808. doi: 10.1002/14651858.CD003808.pub2

49. van ’t Hof MA, Frencken JE, van Palenstein Helderman WH, Holmgren CJ. The atraumatic restorative treatment (ART) approach for managing dental caries: a meta-analysis. *Int Dent J* (2006) 56:345–351. doi: 10.1111/j.1875-595x.2006.tb00339.x
