# Supplementary material for: Effect of minimal intervention on carious lesions in primary teeth. An Umbrella review
Source: Front Dent Med. 2026 Jan 12;6:1751752. doi: 10.3389/fdmed.2025.1751752 (PMC12833399; doi:10.3389/fdmed.2025.1751752)
Supplement: Supplementary file 4 [file Table4.docx]

Supplementary Material 5. Overlapping of primary studies in systematic reviews

| **Primary studies** | **Systematic reviews that included the primary studies** | **Times that primary studies were included** |
| --- | --- | --- |
| Zhi et al. (1) | Bukhari et al. (2), Muntean et al. (3), Vishwanathaiah et al. (4), Tedesco et al. (5), Contreras et al. (6), Chibinski et al. (7), Duangthip et al. (8), Gao et al. (9), Duangthip et al. (10) | 9 |
| Duangthip et al. (11) | Bukhari et al. (2), Muntean et al. (3), Alqalaleef et al. (12), Trieu et al. (13), Tedesco et al. (5), Contreras et al. (6), Chibinski et al. (7), Gao et al. (9) | 8 |
| Dos Santos et al. (14) | Muntean et al. (3), Oliveira et al. (15), Tedesco et al. (5), Contreras et al. (6), Chibinski et al. (7), Duangthip et al. (8), Gao et al. (9), Duangthip et al. (10) | 8 |
| Chu et al. (16) | Vishwanathaiah et al. (4), Jabin et al. (17), Oliveira et al. (15), Trieu et al. (13), Chibinski et al. (7), Gao et al. (9), Duangthip et al. (10), Marinho et al. (18) | 8 |
| Llodra et al. (19) | Bukhari et al. (2), Vishwanathaiah et al. (4), Jabin et al. (17), Oliveira et al. (15), Contreras et al. (6), Chibinski et al. (7), Gao et al. (9) | 7 |
| Honkala et al. (20) | Chaudhari et al. (21), Tedesco et al. (5), Ruengrungsom et al. (22), Tedesco et al. (23), Raggio et al. (24), de Amorim et al. (25), van 't Hof et al. (26) | 7 |
| Ribeiro et al. (27) | Schwendicke et al. (28), Aïem et al. (29), Pedrotti et al. (30), Li et al. (31), Schwendicke et al. (32), Ricketts et al. (33), Ricketts et al. (34) | 7 |
| Phonghanyudh et al. (35) | Schwendicke et al. (28), Santamaría et al. (36), Aïem et al. (29), Pedrotti et al. (30), Li et al. (31), Schwendicke et al. (32) | 6 |
| Liu et al. (37) | Bukhari et al. (2), Muntean et al. (3), Vishwanathaiah et al. (4), Papageorgiou et al. (38), Chibinski et al. (7), Marinho et al. (18) | 6 |
| Yee et al. (39) | Bukhari et al. (2), Vishwanathaiah et al. (4), Jabin et al. (17), Contreras et al. (6), Chibinski et al. (7), Gao et al. (9) | 6 |
| Lula et al. (40) | Schwendicke et al. (28), Aïem et al. (29), Li et al. (31), Schwendicke et al. (32), Ricketts et al. (33), Ferreira et al. (41) | 6 |
| Yu et al. (42) | Chaudhari et al. (21), Tedesco et al. (5), Ruengrungsom et al. (22), Dorri et al. (43), de Amorim et al. (25), van 't Hof et al. (26) | 6 |
| Taifour et al. (44) | Tedesco et al. (5), Ruengrungsom et al. (22), Tedesco et al. (23), Raggio et al. (24), de Amorim et al. (25), van 't Hof et al. (26) | 6 |
| Orhan et al. (45) | Schwendicke et al. (28), Aïem et al. (29), Li et al. (31), Schwendicke et al. (32), Ricketts et al. (33) | 5 |
| Ekstrand et al. (46) | Cebula et al. (47), Tedesco et al. (48), Chen et al. (49), Elrashid et al. (50), Dorri et al. (51) | 5 |
| Ersin et al. (52) | Tedesco et al. (5), Ruengrungsom et al. (22), Tedesco et al. (23), Raggio et al. (24), de Amorim et al. (25) | 5 |
| Foley et al. (53) | Schwendicke et al. (28), Pedrotti et al. (30), Li et al. (31), Schwendicke et al. (32), Ferreira et al. (41) | 5 |
| Magnusson et al. (54) | Schwendicke et al. (28), Aïem et al. (29), Schwendicke et al. (32), Ricketts et al. (33), Ricketts et al. (34) | 5 |
| Gao et al. (55) | Bukhari et al. (2), Muntean et al. (3), Vishwanathaiah et al. (4), Alqalaleef et al. (12) | 4 |
| Mabangkhru et al. (56) | Bukhari et al. (2), Muntean et al. (3), Vishwanathaiah et al. (4), Alqalaleef et al. (12) | 4 |
| Jorge et al. (57) | Tasleem et al. (58), Cebula et al. (47), Tedesco et al. (48), Chen et al. (49) | 4 |
| Tirupathi et al. (59) | Bukhari et al. (2), Muntean et al. (3), Vishwanathaiah et al. (4), Alqalaleef et al. (12) | 4 |
| Bagher et al. (60) | Tasleem et al. (58), Cebula et al. (47), Tedesco et al. (48), Chen et al. (49) | 4 |
| Duangthip et al. (61) | Bukhari et al. (2), Muntean et al. (3), Alqalaleef et al. (12), Trieu et al. (13) | 4 |
| Arthur et al. (62) | Tasleem et al. (58), Cebula et al. (47), Chen et al. (49), Elrashid et al. (50) | 4 |
| Foster Page et al. (63) | Tasleem et al. (58), Cebula et al. (47), Chen et al. (49), Elrashid et al. (50) | 4 |
| Meyer-Lueckel et al. (64) | Tasleem et al. (58), Cebula et al. (47), Chen et al. (49), Elrashid et al. (50) | 4 |
| Monse et al. (65) | Muntean et al. (3), Vishwanathaiah et al. (4), Contreras et al. (6), Chibinski et al. (7) | 4 |
| Martignon et al. (66) | Cebula et al. (47), Chen et al. (49), Elrashid et al. (50), Dorri et al. (51) | 4 |
| Innes et al. (67) | Hu et al. (68), Schwendicke et al. (28), Tedesco et al. (5), Innes et al. (69) | 4 |
| Leksell et al. (70) | Schwendicke et al. (28), Schwendicke et al. (32), Ricketts et al. (33), Ricketts et al. (34) | 4 |
| Sirivichayakul et al. (71) | Bukhari et al. (2), Alqalaleef et al. (12), Inchingolo et al. (72) | 3 |
| Abdellatif et al. (73) | Bukhari et al. (2), Vishwanathaiah et al. (4), Alqalaleef et al. (12) | 3 |
| Aly et al. (74) | Mohapatra et al. (75), Dipalma et al. (76), Inchingolo et al. (72) | 3 |
| Cleary et al. (77) | Bukhari et al. (2), Muntean et al. (3), Alqalaleef et al. (12) | 3 |
| Phonghanyudh et al. (78) | Bukhari et al. (2), Muntean et al. (3), Alqalaleef et al. (12) | 3 |
| Boyd et al. (79) | Chua et al. (80), Hu et al. (68), Schwendicke et al. (28) | 3 |
| Abdellatif et al. (81) | Bukhari et al. (2), Muntean et al. (3), Alqalaleef et al. (12) | 3 |
| Sarti et al. (82) | Tasleem et al. (58), Cebula et al. (47), Tedesco et al. (48) | 3 |
| Arslan et al. (83) | Chen et al. (49), Tasleem et al. (58), Cebula et al. (47) | 3 |
| Paris et al. (84) | Tasleem et al. (58), Cebula et al. (47), Chen et al. (49) | 3 |
| Elamin et al. (85) | Inchingolo et al. (86), Chua et al. (80), Hu et al. (68) | 3 |
| Vollú et al. (87) | Bukhari et al. (2), Muntean et al. (3), Alqalaleef et al. (12) | 3 |
| Peters et al. (88) | Tasleem et al. (58), Cebula et al. (47), Chen et al. (49) | 3 |
| Ammari et al. (89) | Cebula et al. (47), Chen et al. (49), Elrashid et al. (50) | 3 |
| Fung et al. (90) | Muntean et al. (3), Vishwanathaiah et al. (4), Jabin et al. (17) | 3 |
| Peters et al. (91) | Tasleem et al. (58), Cebula et al. (47), Chen et al. (49) | 3 |
| Franzon et al. (92) | Santamaría et al. (36), Aïem et al. (29), Pedrotti et al. (30) | 3 |
| Mijan et al. (93) | Chaudhari et al. (21), Santamaría et al. (36), Tedesco et al. (5) | 3 |
| Franzon et al. (94) | Schwendicke et al. (28), Aïem et al. (29), Li et al. (31) | 3 |
| Santamaria et al. (95) | Inchingolo et al. (86), Tedesco et al. (5), Innes et al. (69) | 3 |
| Hilgert et al. (96) | Chaudhari et al. (21), Ruengrungsom et al. (22), Tedesco et al. (23) | 3 |
| Meyer-Lueckel et al. (97) | Cebula et al. (47), Chen et al. (49), Elrashid et al. (50) | 3 |
| Martignon et al. (98) | Tedesco et al. (48), Chen et al. (49), Dorri et al. (51) | 3 |
| Paris et al. (99) | Cebula et al. (47), Chen et al. (49), Dorri et al. (51) | 3 |
| Braga et al. (100) | Bukhari et al. (2), Contreras et al. (6), Gao et al. (9) | 3 |
| Gao et al. (101) | Ruengrungsom et al. (22), de Amorim et al. (25), van 't Hof et al. (26) | 3 |
| Louw et al. (102) | Tedesco et al. (5), de Amorim et al. (25), van 't Hof et al. (26) | 3 |
| Ziraps et al. (103) | Ruengrungsom et al. (22), de Amorim et al. (25), van 't Hof et al. (26) | 3 |
| Lo et al. (104) | Ruengrungsom et al. (22), de Amorim et al. (25), van 't Hof et al. (26) | 3 |
| Ho et al. (105) | Ruengrungsom et al. (22), de Amorim et al. (25), van 't Hof et al. (26) | 3 |
| Frencken et al. (106) | Ruengrungsom et al. (22), de Amorim et al. (25), van 't Hof et al. (26) | 3 |
| Hamza et al. (107) | Dipalma et al. (76), Bukhari et al. (2) | 2 |
| Bansal et al. (108) | Mohapatra et al. (75), Inchingolo et al. (72) | 2 |
| Yassin et al. (109) | Bukhari et al. (2), Vishwanathaiah et al. (4) | 2 |
| Zheng et al. (110) | Bukhari et al. (2), Alqalaleef et al. (12) | 2 |
| Patel et al. (111) | Mohapatra et al. (75), Inchingolo et al. (72) | 2 |
| Mohammed et al. (112) | Mohapatra et al. (75), Dipalma et al. (76) | 2 |
| Azuoru et al. (113) | Bukhari et al. (2), Alqalaleef et al. (12) | 2 |
| Mani Prakash et al. (114) | Alqalaleef et al. (12), Inchingolo et al. (72) | 2 |
| Ayedun et al. (115) | Chua et al. (80), Hu et al. (68) | 2 |
| Kaptan et al. (116) | Inchingolo et al. (86), Hu et al. (68) | 2 |
| Turton et al. (117) | Bukhari et al. (2), Muntean et al. (3) | 2 |
| Binladen et al. (118) | Inchingolo et al. (86), Chua et al. (80) | 2 |
| Jiang et al. (119) | Dipalma et al. (76), Muntean et al. (3) | 2 |
| Al-Nerabieah et al. (120) | Bukhari et al. (2), Alqalaleef et al. (12) | 2 |
| Ebrahimi et al. (121) | Chua et al. (80), Hu et al. (68) | 2 |
| Araujo et al. (122) | Hu et al. (68), Schwendicke et al. (28) | 2 |
| Santamaria et al. (123) | Hu et al. (68), Schwendicke et al. (28) | 2 |
| Mello et al. (124) | Schwendicke et al. (28), Aïem et al. (29) | 2 |
| Fracasso et al. (125) | Bukhari et al. (2), Inchingolo et al. (72) | 2 |
| Basili et al. (126) | Tasleem et al. (58), Chen et al. (49) | 2 |
| Qvist et al. (127) | Schwendicke et al. (28), Papageorgiou et al. (38) | 2 |
| Hesse et al. (128) | Inchingolo et al. (86), Dipalma et al. (76) | 2 |
| Hesse et al. (129) | Dipalma et al. (76), Ruengrungsom et al. (22) | 2 |
| de Amorin et al. (130) | Chaudhari et al. (21), Ruengrungsom et al. (22) | 2 |
| Hesse et al. (131) | Schwendicke et al. (28), Tedesco et al. (5) | 2 |
| Karaman et al. (132) | Pagano et al. (133), Papageorgiou et al. (38) | 2 |
| Borges et al. (134) | Schwendicke et al. (28), Tedesco et al. (5) | 2 |
| Bohari et al. (135) | Deng et al. (136), Lai et al. (137) | 2 |
| Alkilzy et al. (138) | Chen et al. (49), Dorri et al. (51) | 2 |
| Trairatvorakul et al. (139) | Chen et al. (49), Dorri et al. (51) | 2 |
| Bjørndal et al. (140) | Schwendicke et al. (32), Ricketts et al. (33) | 2 |
| Farag et al. (141) | Ruengrungsom et al. (22), de Amorim et al. (25) | 2 |
| Ercan et al. (142) | Ruengrungsom et al. (22), de Amorim et al. (25) | 2 |
| Frencken et al. (143) | Chaudhari et al. (21), Ruengrungsom et al. (22) | 2 |
| Baca et al. (144) | Ramamurthy et al. (145), Papageorgiou et al. (38) | 2 |
| Innes et al. (146) | Ricketts et al. (33), Ferreira et al. (41) | 2 |
| van Gemert-Schriks et al. (147) | Ruengrungsom et al. (22), de Amorim et al. (25) | 2 |
| Lo et al. (148) | Ruengrungsom et al. (22), de Amorim et al. (25) | 2 |
| Lozano-Chourio et al. (149) | Deng et al. (136), Lai et al. (137) | 2 |
| Martignon et al. (150) | Chen et al. (49), Dorri et al. (51) | 2 |
| Menezes et al. (151) | Duangthip et al. (8), de Amorim et al. (25) | 2 |
| Frencken et al. (152) | de Amorim et al. (25), van 't Hof et al. (26) | 2 |
| Corona et al. (153) | Ramamurthy et al. (145), Papageorgiou et al. (38) | 2 |
| Gomez et al. (154) | Chen et al. (49), Dorri et al. (51) | 2 |
| van den Dungen et al. (155) | Tedesco et al. (5), Dorri et al. (43) | 2 |
| Loh et al. (156) | de Amorim et al. (25), van 't Hof et al. (26) | 2 |
| Mandari et al. (157) | Ruengrungsom et al. (22), van 't Hof et al. (26) | 2 |
| Kalf-Scholte et al. (158) | Ruengrungsom et al. (22), van 't Hof et al. (26) | 2 |
| Rahimtoola et al. (159) | de Amorim et al. (25), van 't Hof et al. (26) | 2 |
| Yip et al. (160) | de Amorim et al. (25), van 't Hof et al. (26) | 2 |
| Yip et al. (161) | de Amorim et al. (25), van 't Hof et al. (26) | 2 |
| Lo et al. (162) | Vishwanathaiah et al. (4), Trieu et al. (13) | 2 |
| Yee et al. (163) | Ruengrungsom et al. (22), van 't Hof et al. (26) | 2 |
| Kikwilu et al. (164) | de Amorim et al. (25), van 't Hof et al. (26) | 2 |
| Lo et al. (165) | Ruengrungsom et al. (22), van 't Hof et al. (26) | 2 |
| Holmgren et al. (166) | Ruengrungsom et al. (22), van 't Hof et al. (26) | 2 |
| Mickenautsch et al. (167) | de Amorim et al. (25), van 't Hof et al. (26) | 2 |
| Luo et al. (168) | de Amorim et al. (25), van 't Hof et al. (26) | 2 |
| Mertz-Fairhurst et al. (169) | Schwendicke et al. (28), Schwendicke-b | 2 |
| Frencken et al. (170) | Ruengrungsom et al. (22), van 't Hof et al. (26) | 2 |
| Phantumvanit et al. (171) | Ruengrungsom et al. (22), van 't Hof et al. (26) | 2 |
| Mertz-Fairhurst et al. (172) | Ricketts et al. (33), Ricketts et al. (34) | 2 |

**References**

1. Zhi QH, Lo ECM, Lin HC. Randomized clinical trial on effectiveness of silver diamine fluoride and glass ionomer in arresting dentine caries in preschool children. *J Dent* (2012) 40:962–967. doi: 10.1016/j.jdent.2012.08.002

2. Bukhari OM. Effectiveness of topical silver diamine fluoride for management of dental caries in children and early adolescents: A systematic review and meta-analysis. *Rom J Oral Rehabil* (2025) 17:975–992. doi: 10.62610/RJOR.2025.2.17.89

3. Muntean A, Mzoughi SM, Pacurar M, Candrea S, Inchingolo AD, Inchingolo AM, Ferrante L, Dipalma G, Inchingolo F, Palermo A, et al. Silver Diamine Fluoride in Pediatric Dentistry: Effectiveness in Preventing and Arresting Dental Caries-A Systematic Review. *Children (Basel)* (2024) 11:499. doi: 10.3390/children11040499

4. Vishwanathaiah S, Maganur PC, Syed AA, Kakti A, Hussain Jaafari AH, Albar DH, Renugalakshmi A, Jeevanandan G, Khurshid Z, Ali Baeshen H, et al. Effectiveness of silver diamine fluoride (SDF) in arresting coronal dental caries in children and adolescents: a systematic review. *J Clin Pediatr Dent* (2024) 48:27–40. doi: 10.22514/jocpd.2024.101

5. Tedesco TK, Gimenez T, Floriano I, Montagner AF, Camargo LB, Calvo AFB, Morimoto S, Raggio DP. Scientific evidence for the management of dentin caries lesions in pediatric dentistry: A systematic review and network meta-analysis. *PLoS One* (2018) 13:e0206296. doi: 10.1371/journal.pone.0206296

6. Contreras V, Toro MJ, Elías-Boneta AR, Encarnación-Burgos A. Effectiveness of silver diamine fluoride in caries prevention and arrest: a systematic literature review. *Gen Dent* (2017) 65:22–29.

7. Chibinski AC, Wambier LM, Feltrin J, Loguercio AD, Wambier DS, Reis A. Silver Diamine Fluoride Has Efficacy in Controlling Caries Progression in Primary Teeth: A Systematic Review and Meta-Analysis. *Caries Res* (2017) 51:527–541. doi: 10.1159/000478668

8. Duangthip D, Jiang M, Chu CH, Lo ECM. Restorative approaches to treat dentin caries in preschool children: systematic review. *Eur J Paediatr Dent* (2016) 17:113–121.

9. Gao SS, Zhao IS, Hiraishi N, Duangthip D, Mei ML, Lo ECM, Chu CH. Clinical Trials of Silver Diamine Fluoride in Arresting Caries among Children: A Systematic Review. *JDR Clin Trans Res* (2016) 1:201–210. doi: 10.1177/2380084416661474

10. Duangthip D, Jiang M, Chu CH, Lo ECM. Non-surgical treatment of dentin caries in preschool children--systematic review. *BMC Oral Health* (2015) 15:44. doi: 10.1186/s12903-015-0033-7

11. Duangthip D, Chu CH, Lo ECM. A randomized clinical trial on arresting dentine caries in preschool children by topical fluorides--18 month results. *J Dent* (2016) 44:57–63. doi: 10.1016/j.jdent.2015.05.006

12. Alqalaleef SS, Alnakhli RA, Ezzat Y, AlQadi HI, Aljilani AD, Natto ZS. The role of silver diamine fluoride as dental caries preventive and arresting agent: a systematic review and meta-analysis. *Front Oral Health* (2024) 5:1492762. doi: 10.3389/froh.2024.1492762

13. Trieu A, Mohamed A, Lynch E. Silver diamine fluoride versus sodium fluoride for arresting dentine caries in children: a systematic review and meta-analysis. *Sci Rep* (2019) 9:2115. doi: 10.1038/s41598-019-38569-9

14. Dos Santos VE, de Vasconcelos FMN, Ribeiro AG, Rosenblatt A. Paradigm shift in the effective treatment of caries in schoolchildren at risk. *Int Dent J* (2012) 62:47–51. doi: 10.1111/j.1875-595X.2011.00088.x

15. Oliveira BH, Rajendra A, Veitz-Keenan A, Niederman R. The Effect of Silver Diamine Fluoride in Preventing Caries in the Primary Dentition: A Systematic Review and Meta-Analysis. *Caries Res* (2019) 53:24–32. doi: 10.1159/000488686

16. Chu CH, Lo ECM, Lin HC. Effectiveness of silver diamine fluoride and sodium fluoride varnish in arresting dentin caries in Chinese pre-school children. *J Dent Res* (2002) 81:767–770. doi: 10.1177/0810767

17. Jabin Z, Vishnupriya V, Agarwal N, Nasim I, Jain M, Sharma A. Effect of 38% silver diamine fluoride on control of dental caries in primary dentition: A Systematic review. *J Family Med Prim Care* (2020) 9:1302–1307. doi: 10.4103/jfmpc.jfmpc_1017_19

18. Marinho V, Worthington H, Walsh T, Clarkson J. Fluoride varnishes for preventing dental caries in children and adolescents. *Cochrane Database Syst Rev* (2013) 2013:CD002279. doi: 10.1002/14651858.CD002279.pub2

19. Llodra JC, Rodriguez A, Ferrer B, Menardia V, Ramos T, Morato M. Efficacy of silver diamine fluoride for caries reduction in primary teeth and first permanent molars of schoolchildren: 36-month clinical trial. *J Dent Res* (2005) 84:721–724. doi: 10.1177/154405910508400807

20. Honkala E, Behbehani J, Ibricevic H, Kerosuo E, Al-Jame G. The atraumatic restorative treatment (ART) approach to restoring primary teeth in a standard dental clinic. *Int J Paediatr Dent* (2003) 13:172–179. doi: 10.1046/j.1365-263x.2003.00455.x

21. Chaudhari HG, Patil RU, Jathar PN, Jain CA. A systematic review of randomized controlled trials on survival rate of atraumatic restorative treatment compared with conventional treatment on primary dentition. *J Indian Soc Pedod Prev Dent* (2022) 40:112–117. doi: 10.4103/jisppd.jisppd_119_22

22. Ruengrungsom C, Palamara JEA, Burrow MF. Comparison of ART and conventional techniques on clinical performance of glass-ionomer cement restorations in load bearing areas of permanent and primary dentitions: A systematic review. *J Dent* (2018) 78:1–21. doi: 10.1016/j.jdent.2018.07.008

23. Tedesco TK, Calvo AFB, Lenzi TL, Hesse D, Guglielmi CAB, Camargo LB, Gimenez T, Braga MM, Raggio DP. ART is an alternative for restoring occlusoproximal cavities in primary teeth - evidence from an updated systematic review and meta-analysis. *Int J Paediatr Dent* (2017) 27:201–209. doi: 10.1111/ipd.12252

24. Raggio DP, Hesse D, Lenzi TL, Guglielmi CAB, Braga MM. Is Atraumatic restorative treatment an option for restoring occlusoproximal caries lesions in primary teeth? A systematic review and meta-analysis. *Int J Paediatr Dent* (2013) 23:435–443. doi: 10.1111/ipd.12013

25. de Amorim RG, Leal SC, Frencken JE. Survival of atraumatic restorative treatment (ART) sealants and restorations: a meta-analysis. *Clin Oral Investig* (2012) 16:429–441. doi: 10.1007/s00784-011-0513-3

26. van ’t Hof MA, Frencken JE, van Palenstein Helderman WH, Holmgren CJ. The atraumatic restorative treatment (ART) approach for managing dental caries: a meta-analysis. *Int Dent J* (2006) 56:345–351. doi: 10.1111/j.1875-595x.2006.tb00339.x

27. Ribeiro CC, Baratieri LN, Perdigão J, Baratieri NM, Ritter AV. A clinical, radiographic, and scanning electron microscopic evaluation of adhesive restorations on carious dentin in primary teeth. *Quintessence Int* (1999) 30:591–599.

28. Schwendicke F, Walsh T, Lamont T, Al-Yaseen W, Bjørndal L, Clarkson JE, Fontana M, Gomez Rossi J, Göstemeyer G, Levey C, et al. Interventions for treating cavitated or dentine carious lesions. *Cochrane Database Syst Rev* (2021) 7:CD013039. doi: 10.1002/14651858.CD013039.pub2

29. Aïem E, Joseph C, Garcia A, Smaïl-Faugeron V, Muller-Bolla M. Caries removal strategies for deep carious lesions in primary teeth: Systematic review. *Int J Paediatr Dent* (2020) 30:392–404. doi: 10.1111/ipd.12616

30. Pedrotti D, Cavalheiro CP, Casagrande L, de Araújo FB, Pettorossi Imparato JC, de Oliveira Rocha R, Lenzi TL. Does selective carious tissue removal of soft dentin increase the restorative failure risk in primary teeth?: Systematic review and meta-analysis. *J Am Dent Assoc* (2019) 150:582-590.e1. doi: 10.1016/j.adaj.2019.02.018

31. Li T, Zhai X, Song F, Zhu H. Selective versus non-selective removal for dental caries: a systematic review and meta-analysis. *Acta Odontol Scand* (2018) 76:135–140. doi: 10.1080/00016357.2017.1392602

32. Schwendicke F, Dörfer C, Paris S. Incomplete Caries Removal: A Systematic Review and Meta-analysis. *J Dent Res* (2013) 92:306–314. doi: 10.1177/0022034513477425

33. Ricketts D, Lamont T, Innes NPT, Kidd E, Clarkson JE. Operative caries management in adults and children. *Cochrane Database Syst Rev* (2013) 28:CD003808. doi: 10.1002/14651858.CD003808.pub3

34. Ricketts DNJ, Kidd E a. M, Innes N, Clarkson J. Complete or ultraconservative removal of decayed tissue in unfilled teeth. *Cochrane Database Syst Rev* (2006) 19:CD003808. doi: 10.1002/14651858.CD003808.pub2

35. Phonghanyudh A, Phantumvanit P, Songpaisan Y, Petersen PE. Clinical evaluation of three caries removal approaches in primary teeth: a randomised controlled trial. *Community Dent Health* (2012) 29:173–178.

36. Santamaría RM, Abudrya MH, Gul G, Mourad MS, Felix Gomez GF, Ferreira Zandona AGF. How to Intervene in the Caries Process: Dentin Caries in Primary Teeth. *Caries Res* (2020) 54:306–323. doi: 10.1159/000508899

37. Liu BY, Lo ECM, Chu CH, Lin HC. Randomized trial on fluorides and sealants for fissure caries prevention. *J Dent Res* (2012) 91:753–758. doi: 10.1177/0022034512452278

38. Papageorgiou SN, Dimitraki D, Kotsanos N, Bekes K, van Waes H. Performance of pit and fissure sealants according to tooth characteristics: A systematic review and meta-analysis. *J Dent* (2017) 66:8–17. doi: 10.1016/j.jdent.2017.08.004

39. Yee R, Holmgren C, Mulder J, Lama D, Walker D, van Palenstein Helderman W. Efficacy of silver diamine fluoride for Arresting Caries Treatment. *J Dent Res* (2009) 88:644–647. doi: 10.1177/0022034509338671

40. Lula ECO, Monteiro-Neto V, Alves CMC, Ribeiro CCC. Microbiological analysis after complete or partial removal of carious dentin in primary teeth: a randomized clinical trial. *Caries Res* (2009) 43:354–358. doi: 10.1159/000231572

41. Ferreira JMS, Pinheiro SL, Sampaio FC, de Menezes VA. Caries removal in primary teeth--a systematic review. *Quintessence Int* (2012) 43:e9-15.

42. Yu C, Gao XJ, Deng DM, Yip HK, Smales RJ. Survival of glass ionomer restorations placed in primary molars using atraumatic restorative treatment (ART) and conventional cavity preparations: 2-year results. *Int Dent J* (2004) 54:42–46. doi: 10.1111/j.1875-595x.2004.tb00251.x

43. Dorri M, Martinez-Zapata MJ, Walsh T, Marinho VC, Sheiham Deceased A, Zaror C. Atraumatic restorative treatment versus conventional restorative treatment for managing dental caries. *Cochrane Database Syst Rev* (2017) 12:CD008072. doi: 10.1002/14651858.CD008072.pub2

44. Taifour D, Frencken JE, Beiruti N, van ’t Hof MA, Truin GJ. Effectiveness of glass-ionomer (ART) and amalgam restorations in the deciduous dentition: results after 3 years. *Caries Res* (2002) 36:437–444. doi: 10.1159/000066531

45. Orhan AI, Oz FT, Orhan K. Pulp exposure occurrence and outcomes after 1- or 2-visit indirect pulp therapy vs complete caries removal in primary and permanent molars. *Pediatr Dent* (2010) 32:347–355.

46. Ekstrand KR, Bakhshandeh A, Martignon S. Treatment of proximal superficial caries lesions on primary molar teeth with resin infiltration and fluoride varnish versus fluoride varnish only: efficacy after 1 year. *Caries Res* (2010) 44:41–46. doi: 10.1159/000275573

47. Cebula M, Göstemeyer G, Krois J, Pitchika V, Paris S, Schwendicke F, Effenberger S. Resin Infiltration of Non-Cavitated Proximal Caries Lesions in Primary and Permanent Teeth: A Systematic Review and Scenario Analysis of Randomized Controlled Trials. *J Clin Med* (2023) 12:727. doi: 10.3390/jcm12020727

48. Tedesco TK, Calvo AFB, Pássaro AL, Araujo MP, Ladewig NM, Scarpini S, Lara JS, Braga MM, Gimenez T, Raggio DP. Nonrestorative treatment of initial caries lesion in primary teeth: a systematic review and network meta-analysis. *Acta Odontol Scand* (2022) 80:1–8. doi: 10.1080/00016357.2021.1928748

49. Chen Y, Chen D, Lin H. Infiltration and sealing for managing non-cavitated proximal lesions: a systematic review and meta-analysis. *BMC Oral Health* (2021) 21:13. doi: 10.1186/s12903-020-01364-4

50. Elrashid AH, Alshaiji BS, Saleh SA, Zada KA, Baseer MA. Efficacy of Resin Infiltrate in Noncavitated Proximal Carious Lesions: A Systematic Review and Meta-Analysis. *J Int Soc Prev Community Dent* (2019) 9:211–218. doi: 10.4103/jispcd.JISPCD_26_19

51. Dorri M, Dunne SM, Walsh T, Schwendicke F. Micro-invasive interventions for managing proximal dental decay in primary and permanent teeth. *Cochrane Database Syst Rev* (2015) 2015:CD010431. doi: 10.1002/14651858.CD010431.pub2

52. Ersin NK, Candan U, Aykut A, Onçağ O, Eronat C, Kose T. A clinical evaluation of resin-based composite and glass ionomer cement restorations placed in primary teeth using the ART approach: results at 24 months. *J Am Dent Assoc* (2006) 137:1529–1536. doi: 10.14219/jada.archive.2006.0087

53. Foley J, Evans D, Blackwell A. Partial caries removal and cariostatic materials in carious primary molar teeth: a randomised controlled clinical trial. *Br Dent J* (2004) 197:697–701; discussion 689. doi: 10.1038/sj.bdj.4811865

54. Magnusson BO, Sundell SO. Stepwise excavation of deep carious lesions in primary molars. *J Int Assoc Dent Child* (1977) 8:36–40.

55. Gao SS, Chen KJ, Duangthip D, Wong MCM, Lo ECM, Chu CH. Arresting early childhood caries using silver and fluoride products - A randomised trial. *J Dent* (2020) 103:103522. doi: 10.1016/j.jdent.2020.103522

56. Mabangkhru S, Duangthip D, Chu CH, Phonghanyudh A, Jirarattanasopha V. A randomized clinical trial to arrest dentin caries in young children using silver diamine fluoride. *J Dent* (2020) 99:103375. doi: 10.1016/j.jdent.2020.103375

57. Jorge RC, Ammari MM, Soviero VM, Souza IPR. Randomized controlled clinical trial of resin infiltration in primary molars: 2 years follow-up. *J Dent* (2019) 90:103184. doi: 10.1016/j.jdent.2019.103184

58. Tasleem R, Alqahtani SA, Abogazalah N, Almubarak H, Riaz A, Ali SS, Allana Z. Microinvasive interventions in the management of proximal caries lesions in primary and permanent teeth- systematic review and meta-analysis. *BMC Oral Health* (2025) 25:48. doi: 10.1186/s12903-024-05400-5

59. Tirupathi S, Svsg N, Rajasekhar S, Nuvvula S. Comparative cariostatic efficacy of a novel Nano-silver fluoride varnish with 38% silver diamine fluoride varnish a double-blind randomized clinical trial. *J Clin Exp Dent* (2019) 11:e105–e112. doi: 10.4317/jced.54995

60. Bagher SM, Hegazi FM, Finkelman M, Ramesh A, Gowharji N, Swee G, Felemban O, Loo CY. Radiographic Effectiveness of Resin Infiltration in Arresting Incipient Proximal Enamel Lesions in Primary Molars. *Pediatr Dent* (2018) 40:195–200.

61. Duangthip D, Wong MCM, Chu CH, Lo ECM. Caries arrest by topical fluorides in preschool children: 30-month results. *J Dent* (2018) 70:74–79. doi: 10.1016/j.jdent.2017.12.013

62. Arthur RA, Zenkner JE, d’Ornellas Pereira Júnior JC, Correia RT, Alves LS, Maltz M. Proximal carious lesions infiltration-a 3-year follow-up study of a randomized controlled clinical trial. *Clin Oral Investig* (2018) 22:469–474. doi: 10.1007/s00784-017-2135-x

63. Foster Page LA, Beckett D, Ahmadi R, Schwass DR, Leon de la Barra S, Moffat SM, Meldrum A, Thomson WM. Resin Infiltration of Caries in Primary Molars in a Community Setting: 24-Month Randomized Controlled Trial Findings. *JDR Clin Trans Res* (2017) 2:287–294. doi: 10.1177/2380084417699400

64. Meyer-Lueckel H, Balbach A, Schikowsky C, Bitter K, Paris S. Pragmatic RCT on the Efficacy of Proximal Caries Infiltration. *J Dent Res* (2016) 95:531–536. doi: 10.1177/0022034516629116

65. Monse B, Heinrich-Weltzien R, Mulder J, Holmgren C, van Palenstein Helderman WH. Caries preventive efficacy of silver diammine fluoride (SDF) and ART sealants in a school-based daily fluoride toothbrushing program in the Philippines. *BMC Oral Health* (2012) 12:52. doi: 10.1186/1472-6831-12-52

66. Martignon S, Ekstrand KR, Gomez J, Lara JS, Cortes A. Infiltrating/sealing proximal caries lesions: a 3-year randomized clinical trial. *J Dent Res* (2012) 91:288–292. doi: 10.1177/0022034511435328

67. Innes NPT, Evans DJP, Stirrups DR. Sealing caries in primary molars: randomized control trial, 5-year results. *J Dent Res* (2011) 90:1405–1410. doi: 10.1177/0022034511422064

68. Hu S, BaniHani A, Nevitt S, Maden M, Santamaria RM, Albadri S. Hall technique for primary teeth: A systematic review and meta-analysis. *Jpn Dent Sci Rev* (2022) 58:286–297. doi: 10.1016/j.jdsr.2022.09.003

69. Innes NPT, Ricketts D, Chong LY, Keightley AJ, Lamont T, Santamaría RM. Preformed crowns for decayed primary molar teeth. *Cochrane Database Syst Rev* (2015) 2015:CD005512. doi: 10.1002/14651858.CD005512.pub3

70. Leksell E, Ridell K, Cvek M, Mejàre I. Pulp exposure after stepwise versus direct complete excavation of deep carious lesions in young posterior permanent teeth. *Endod Dent Traumatol* (1996) 12:192–196. doi: 10.1111/j.1600-9657.1996.tb00513.x

71. Sirivichayakul P, Jirarattanasopha V, Phonghanyudh A, Tunlayadechanont P, Khumsub P, Duangthip D. The effectiveness of topical fluoride agents on preventing development of approximal caries in primary teeth: a randomized clinical trial. *BMC Oral Health* (2023) 23:349. doi: 10.1186/s12903-023-03045-4

72. Inchingolo F, Inchingolo AD, Latini G, Sardano R, Riccaldo L, Mancini A, Palermo A, Inchingolo AM, Dipalma G. Caries in primary molars: is silver diamine fluoride effective in prevention and treatment? A systematic review. *Appl Sci* (2024) 14:2055. doi: 10.3390/app14052055

73. Abdellatif EB, El Kashlan MK, El Tantawi M. Silver diamine fluoride with sodium fluoride varnish versus silver diamine fluoride in arresting early childhood caries: a 6-months follow up of a randomized field trial. *BMC Oral Health* (2023) 23:875. doi: 10.1186/s12903-023-03597-5

74. Aly AAM, Aziz AMA, Elghazawy RK, El Fadl RKA. Survival Analysis and Cost Effectiveness of Silver Modified Atraumatic Restorative Treatment (SMART) and ART Occlusal Restorations in Primary Molars: a randomized controlled trial. *J Dent* (2023) 128:104379. doi: 10.1016/j.jdent.2022.104379

75. Mohapatra S, Mohandas R. Clinical Outcome Success of Silver-Modified Atraumatic Restorative Treatment (SMART) in Treating Children with Dental Caries in Primary Teeth: A Systematic Review. *JHASNU* (2025) 15:4–10. doi: 10.1055/s-0044-1788659

76. Dipalma G, Inchingolo AM, Casamassima L, Nardelli P, Ciccarese D, De Sena P, Inchingolo F, Palermo A, Severino M, Maspero CMN, et al. Effectiveness of Dental Restorative Materials in the Atraumatic Treatment of Carious Primary Teeth in Pediatric Dentistry: A Systematic Review. *Children (Basel)* (2025) 12:511. doi: 10.3390/children12040511

77. Cleary J, Al-Hadidi R, Scully A, Yahn W, Zaid Z, Boynton JR, Eckert GJ, Yanca E, Fontana M. A 12-Month Randomized Clinical Trial of 38% SDF vs. Restorative Treatment. *JDR Clin Trans Res* (2022) 7:135–144. doi: 10.1177/23800844211072741

78. Phonghanyudh A, Duangthip D, Mabangkhru S, Jirarattanasopha V. Is Silver Diamine Fluoride Effective in Arresting Enamel Caries? A Randomized Clinical Trial. *Int J Environ Res Public Health* (2022) 19:8992. doi: 10.3390/ijerph19158992

79. Boyd DH, Thomson WM, Leon de la Barra S, Fuge KN, van den Heever R, Butler BM, Leov F, Foster Page LA. A Primary Care Randomized Controlled Trial of Hall and Conventional Restorative Techniques. *JDR Clin Trans Res* (2021) 6:205–212. doi: 10.1177/2380084420933154

80. Chua DR, Tan BL, Nazzal H, Srinivasan N, Duggal MS, Tong HJ. Outcomes of preformed metal crowns placed with the conventional and Hall techniques: A systematic review and meta-analysis. *Int J Paediatr Dent* (2023) 33:141–157. doi: 10.1111/ipd.13029

81. Abdellatif HM, Ali AM, Baghdady SI, ElKateb MA. Caries arrest effectiveness of silver diamine fluoride compared to alternative restorative technique: randomized clinical trial. *Eur Arch Paediatr Dent* (2021) 22:575–585. doi: 10.1007/s40368-020-00592-0

82. Sarti CS, Vizzotto MB, Filgueiras LV, Bonifácio CC, Rodrigues JA. Two-Year Split-Mouth Randomized Controlled Clinical Trial on the Progression of Proximal Carious Lesions on Primary Molars After Resin Infiltration. *Pediatr Dent* (2020) 42:110–115.

83. Arslan S, Kaplan MH. The Effect of Resin Infiltration on the Progression of Proximal Caries Lesions: A Randomized Clinical Trial. *Med Princ Pract* (2020) 29:238–243. doi: 10.1159/000503053

84. Paris S, Bitter K, Krois J, Meyer-Lueckel H. Seven-year-efficacy of proximal caries infiltration - Randomized clinical trial. *J Dent* (2020) 93:103277. doi: 10.1016/j.jdent.2020.103277

85. Elamin F, Abdelazeem N, Salah I, Mirghani Y, Wong F. A randomized clinical trial comparing Hall vs conventional technique in placing preformed metal crowns from Sudan. *PLoS One* (2019) 14:e0217740. doi: 10.1371/journal.pone.0217740

86. Inchingolo AM, Inchingolo AD, Morolla R, Riccaldo L, Guglielmo M, Palumbo I, Palermo A, Francesco F, Dipalma G. Pre-formed crowns and pediatric dentistry: a systematic review of different techniques of restorations. *J Clin Pediatr Dent* (2025) 49:1–13. doi: 10.22514/jocpd.2025.001

87. Vollú AL, Rodrigues GF, Rougemount Teixeira RV, Cruz LR, Dos Santos Massa G, de Lima Moreira JP, Luiz RR, Barja-Fidalgo F, Fonseca-Gonçalves A. Efficacy of 30% silver diamine fluoride compared to atraumatic restorative treatment on dentine caries arrestment in primary molars of preschool children: A 12-months parallel randomized controlled clinical trial. *J Dent* (2019) 88:103165. doi: 10.1016/j.jdent.2019.07.003

88. Peters MC, Hopkins AR, Zhu L, Yu Q. Efficacy of Proximal Resin Infiltration on Caries Inhibition: Results from a 3-Year Randomized Controlled Clinical Trial. *J Dent Res* (2019) 98:1497–1502. doi: 10.1177/0022034519876853

89. Ammari MM, Jorge RC, Souza IPR, Soviero VM. Efficacy of resin infiltration of proximal caries in primary molars: 1-year follow-up of a split-mouth randomized controlled clinical trial. *Clin Oral Investig* (2018) 22:1355–1362. doi: 10.1007/s00784-017-2227-7

90. Fung MHT, Duangthip D, Wong MCM, Lo ECM, Chu CH. Randomized Clinical Trial of 12% and 38% Silver Diamine Fluoride Treatment. *J Dent Res* (2018) 97:171–178. doi: 10.1177/0022034517728496

91. Peters MC, Hopkins AR, Yu Q. Resin infiltration: An effective adjunct strategy for managing high caries risk-A within-person randomized controlled clinical trial. *J Dent* (2018) 79:24–30. doi: 10.1016/j.jdent.2018.09.005

92. Franzon R, Opdam NJ, Guimarães LF, Demarco FF, Casagrande L, Haas AN, Araujo FB. Randomized controlled clinical trial of the 24-months survival of composite resin restorations after one-step incomplete and complete excavation on primary teeth. *J Dent* (2015) 43:1235–1241. doi: 10.1016/j.jdent.2015.07.011

93. Mijan M, de Amorim RG, Leal SC, Mulder J, Oliveira L, Creugers NHJ, Frencken JE. The 3.5-year survival rates of primary molars treated according to three treatment protocols: a controlled clinical trial. *Clin Oral Investig* (2014) 18:1061–1069. doi: 10.1007/s00784-013-1077-1

94. Franzon R, Guimarães LF, Magalhães CE, Haas AN, Araujo FB. Outcomes of one-step incomplete and complete excavation in primary teeth: a 24-month randomized controlled trial. *Caries Res* (2014) 48:376–383. doi: 10.1159/000357628

95. Santamaria RM, Innes NPT, Machiulskiene V, Evans DJP, Splieth CH. Caries management strategies for primary molars: 1-yr randomized control trial results. *J Dent Res* (2014) 93:1062–1069. doi: 10.1177/0022034514550717

96. Hilgert LA, de Amorim RG, Leal SC, Mulder J, Creugers NHJ, Frencken JE. Is high-viscosity glass-ionomer-cement a successor to amalgam for treating primary molars? *Dent Mater* (2014) 30:1172–1178. doi: 10.1016/j.dental.2014.07.010

97. Meyer-Lueckel H, Bitter K, Paris S. Randomized controlled clinical trial on proximal caries infiltration: three-year follow-up. *Caries Res* (2012) 46:544–548. doi: 10.1159/000341807

98. Martignon S, Tellez M, Santamaría RM, Gomez J, Ekstrand KR. Sealing distal proximal caries lesions in first primary molars: efficacy after 2.5 years. *Caries Res* (2010) 44:562–570. doi: 10.1159/000321986

99. Paris S, Hopfenmuller W, Meyer-Lueckel H. Resin infiltration of caries lesions: an efficacy randomized trial. *J Dent Res* (2010) 89:823–826. doi: 10.1177/0022034510369289

100. Braga MM, Mendes FM, De Benedetto MS, Imparato JCP. Effect of silver diammine fluoride on incipient caries lesions in erupting permanent first molars: a pilot study. *J Dent Child (Chic)* (2009) 76:28–33.

101. Gao W, Peng D, Smales RJ, Yip KHK. Comparison of atraumatic restorative treatment and conventional restorative procedures in a hospital clinic: evaluation after 30 months. *Quintessence Int* (2003) 34:31–37.

102. Louw AJ, Sarvan I, Chikte UME, Honkala E. One-year evaluation of atraumatic restorative treatment and minimum intervention techniques on primary teeth. *SADJ* (2002) 57:366–371.

103. Ziraps A, Honkala E. Clinical trial of a new glass ionomer for an atraumatic restorative treatment technique in class I restorations placed in Latvian school children. *Med Princ Pract* (2002) 11 Suppl 1:44–47. doi: 10.1159/000057778

104. Lo EC, Luo Y, Fan MW, Wei SH. Clinical investigation of two glass-ionomer restoratives used with the atraumatic restorative treatment approach in China: two-years results. *Caries Res* (2001) 35:458–463. doi: 10.1159/000047490

105. Ho TF, Smales RJ, Fang DT. A 2-year clinical study of two glass ionomer cements used in the atraumatic restorative treatment (ART) technique. *Community Dent Oral Epidemiol* (1999) 27:195–201. doi: 10.1111/j.1600-0528.1999.tb02010.x

106. Frencken JE, Makoni F, Sithole WD. ART restorations and glass ionomer sealants in Zimbabwe: survival after 3 years. *Community Dent Oral Epidemiol* (1998) 26:372–381. doi: 10.1111/j.1600-0528.1998.tb01975.x

107. Hamza BE, Attia NM, Abdellatif AM, Hegazy SA. Arresting Active Carious Lesions Using Minimal Intervention Dentistry among a Group of Preschool Children: A Randomized Controlled Clinical Trial. *Int J Clin Pediatr Dent* (2024) 17:1018–1024. doi: 10.5005/jp-journals-10005-2927

108. Bansal K, Shamoo A, Mani K, K PD, Verma A, Mathur VP, Tewari N. Silver diamine fluoride modified atraumatic restorative treatment compared to conventional restorative technique on carious primary molars-A randomized controlled trial. *J Dent* (2023) 138:104698. doi: 10.1016/j.jdent.2023.104698

109. Yassin R, Amer H, Tantawi ME. Effectiveness of silver diamine fluoride versus sodium fluoride varnish combined with mother’s motivational interviewing for arresting early childhood caries: a randomized clinical trial. *BMC Oral Health* (2023) 23:710. doi: 10.1186/s12903-023-03456-3

110. Zheng FM, Yan IG, Duangthip D, Lo ECM, Gao SS, Chu CH. Caries Prevention Using Silver Diamine Fluoride: A 12-Month Clinical Trial. *Int Dent J* (2023) 73:667–673. doi: 10.1016/j.identj.2022.12.005

111. Patel MC, Makwani DA, Bhatt RK, Raj V, Patel C, Patel F. Evaluation of silver-modified atraumatic restorative technique versus conventional pulp therapy in asymptomatic deep carious lesion of primary molars - A comparative prospective clinical study. *J Indian Soc Pedod Prev Dent* (2022) 40:383–390. doi: 10.4103/jisppd.jisppd_360_22

112. Mohammed SME, Awad SM, Wahba AH. Comparison of Clinical Outcomes of Silver-modified Atraumatic Restorative Technique vs Atraumatic Restorative Technique in Primary Teeth: A Randomized Controlled Trial. *J Contemp Dent Pract* (2022) 23:1140–1145. doi: 10.5005/jp-journals-10024-3437

113. Azuoru MO, Ashiwaju MO, Edomwonyi A, Oyapero A, Obisesan B, Omotuyole A. Randomized controlled trial on the effectiveness of silver diamine fluoride in arresting caries in Lagos, Nigeria. *Braz J Oral Sci* (2022) 21:e226341. doi: https://doi.org/10.20396/bjos.v21i00.8666341

114. Mani Prakash DK, Vinay C, Uloopi KS, RojaRamya KS, Penmatsa C, Chandana N. Evaluation of caries arresting potential of silver diamine fluoride and sodium fluoride varnish in primary molars: A randomized controlled trial. *J Indian Soc Pedod Prev Dent* (2022) 40:377–382. doi: 10.4103/jisppd.jisppd_239_22

115. Ayedun OS, Oredugba FA, Sote EO. Comparison of the treatment outcomes of the conventional stainless steel crown restorations and the hall technique in the treatment of carious primary molars. *Niger J Clin Pract* (2021) 24:584–594. doi: 10.4103/njcp.njcp_460_20

116. Kaptan A, Korkmaz E. Evaluation of success of stainless steel crowns placed using the hall technique in children with high caries risk: A randomized clinical trial. *Niger J Clin Pract* (2021) 24:425–434. doi: 10.4103/njcp.njcp_112_20

117. Turton B, Horn R, Durward C. Caries arrest and lesion appearance using two different silver fluoride therapies on primary teeth with and without potassium iodide: 12-month results. *Clin Exp Dent Res* (2021) 7:609–619. doi: 10.1002/cre2.367

118. Binladen H, Al Halabi M, Kowash M, Al Salami A, Khamis AH, Hussein I. A 24-month retrospective study of preformed metal crowns: the Hall technique versus the conventional preparation method. *Eur Arch Paediatr Dent* (2021) 22:67–75. doi: 10.1007/s40368-020-00528-8

119. Jiang M, Wong MCM, Chu CH, Dai L, Lo ECM. A 24-month randomized controlled trial on the success rates of restoring untreated and SDF-treated dentine caries lesions in primary teeth with the ART approach. *J Dent* (2020) 100:103435. doi: 10.1016/j.jdent.2020.103435

120. Al-Nerabieah Z, Arrag E, Rajab A. Cariostatic efficacy and children acceptance of nano-silver fluoride versus silver diamine fluoride: a randomized controlled clinical trial. *J Stoma* (2020) 73:100–106. doi: 10.5114/jos.2020.96939

121. Ebrahimi M, Shirazi AS, Afshari E. Success and Behavior During Atraumatic Restorative Treatment, the Hall Technique, and the Stainless Steel Crown Technique for Primary Molar Teeth. *Pediatr Dent* (2020) 42:187–192.

122. Araujo MP, Innes NP, Bonifácio CC, Hesse D, Olegário IC, Mendes FM, Raggio DP. Atraumatic restorative treatment compared to the Hall Technique for occluso-proximal carious lesions in primary molars; 36-month follow-up of a randomised control trial in a school setting. *BMC Oral Health* (2020) 20:318. doi: 10.1186/s12903-020-01298-x

123. Santamaría RM, Innes NPT, Machiulskiene V, Schmoeckel J, Alkilzy M, Splieth CH. Alternative Caries Management Options for Primary Molars: 2.5-Year Outcomes of a Randomised Clinical Trial. *Caries Res* (2017) 51:605–614. doi: 10.1159/000477855

124. Mello B, C Stafuzza T, Vitor L, Rios D, Silva T, Machado M, M Oliveira T. Evaluation of Dentin-Pulp Complex Response after Conservative Clinical Procedures in Primary Teeth. *Int J Clin Pediatr Dent* (2018) 11:188–192. doi: 10.5005/jp-journals-10005-1509

125. Fracasso M, Venante H, Santin G, Salles C, Provenzano M, Maciel S. Performance of Preventive Methods Applied to the Occlusal Surface of Primary Teeth: A Randomized Clinical Study. *Pesqui Bras Odontopediatria Clín Integr* (2018) 18:1–11. doi: 10.4034/PBOCI.2018.181.05

126. Basili CP, Emilson CG, Corvalan GC, Moran MP, Torres C, Quiroz MD, Gomez SS. Preventive and Therapeutic Proximal Sealing: A 3.5-Year Randomized Controlled Clinical Trial Follow-Up. *Caries Res* (2017) 51:387–393. doi: 10.1159/000470851

127. Qvist V, Borum MK, Møller KD, Andersen TR, Blanche P, Bakhshandeh A. Sealing Occlusal Dentin Caries in Permanent Molars: 7-Year Results of a Randomized Controlled Trial. *JDR Clin Trans Res* (2017) 2:73–86. doi: 10.1177/2380084416680191

128. Hesse D, de Araujo MP, Olegário IC, Innes N, Raggio DP, Bonifácio CC. Atraumatic Restorative Treatment compared to the Hall Technique for occluso-proximal cavities in primary molars: study protocol for a randomized controlled trial. *Trials* (2016) 17:169. doi: 10.1186/s13063-016-1270-z

129. Hesse D, Bonifácio CC, Guglielmi C de AB, Bönecker M, van Amerongen WE, Raggio DP. Bilayer technique and nano-filled coating increase success of approximal ART restorations: a randomized clinical trial. *Int J Paediatr Dent* (2016) 26:231–239. doi: 10.1111/ipd.12194

130. de Amorim RG, Leal SC, Mulder J, Creugers NHJ, Frencken JE. Amalgam and ART restorations in children: a controlled clinical trial. *Clin Oral Investig* (2014) 18:117–124. doi: 10.1007/s00784-013-0955-x

131. Hesse D, Bonifácio CC, Mendes FM, Braga MM, Imparato JCP, Raggio DP. Sealing versus partial caries removal in primary molars: a randomized clinical trial. *BMC Oral Health* (2014) 14:58. doi: 10.1186/1472-6831-14-58

132. Karaman E, Yazici AR, Baseren M, Gorucu J. Comparison of acid versus laser etching on the clinical performance of a fissure sealant: 24-month results. *Oper Dent* (2013) 38:151–158. doi: 10.2341/11-435-C

133. Pagano S, Lombardo G, Orso M, Abraha I, Capobianco B, Cianetti S. Lasers to prevent dental caries: a systematic review. *BMJ Open* (2020) 10:e038638. doi: 10.1136/bmjopen-2020-038638

134. Borges BCD, De Souza Bezerra Araújo RF, Dantas RF, De Araújo Lucena A, De Assunção Pinheiro IV. Efficacy of a non-drilling approach to manage non-cavitated dentin occlusal caries in primary molars: a 12-month randomized controlled clinical trial. *Int J Paediatr Dent* (2012) 22:44–51. doi: 10.1111/j.1365-263X.2011.01156.x

135. Bohari MR, Chunawalla YK, Ahmed BMN. Clinical evaluation of caries removal in primary teeth using conventional, chemomechanical and laser technique: an in vivo study. *J Contemp Dent Pract* (2012) 13:40–47. doi: 10.5005/jp-journals-10024-1093

136. Deng Y, Feng G, Hu B, Kuang Y, Song J. Effects of Papacarie on children with dental caries in primary teeth: a systematic review and meta-analysis. *Int J Paediatr Dent* (2018) 28:361–372. doi: 10.1111/ipd.12364

137. Lai G, Lara Capi C, Cocco F, Cagetti MG, Lingström P, Almhöjd U, Campus G. Comparison of Carisolv system vs traditional rotating instruments for caries removal in the primary dentition: A systematic review and meta-analysis. *Acta Odontol Scand* (2015) 73:569–580. doi: 10.3109/00016357.2015.1023353

138. Alkilzy M, Berndt C, Splieth CH. Sealing proximal surfaces with polyurethane tape: three-year evaluation. *Clin Oral Investig* (2011) 15:879–884. doi: 10.1007/s00784-010-0457-z

139. Trairatvorakul C, Itsaraviriyakul S, Wiboonchan W. Effect of glass-ionomer cement on the progression of proximal caries. *J Dent Res* (2011) 90:99–103. doi: 10.1177/0022034510381265

140. Bjørndal L, Reit C, Bruun G, Markvart M, Kjaeldgaard M, Näsman P, Thordrup M, Dige I, Nyvad B, Fransson H, et al. Treatment of deep caries lesions in adults: randomized clinical trials comparing stepwise vs. direct complete excavation, and direct pulp capping vs. partial pulpotomy. *Eur J Oral Sci* (2010) 118:290–297. doi: 10.1111/j.1600-0722.2010.00731.x

141. Farag A, van der Sanden WJM, Abdelwahab H, Mulder J, Frencken JE. 5-Year survival of ART restorations with and without cavity disinfection. *J Dent* (2009) 37:468–474. doi: 10.1016/j.jdent.2009.03.002

142. Ercan E, Dülgergil CT, Soyman M, Dalli M, Yildirim I. A field-trial of two restorative materials used with atraumatic restorative treatment in rural Turkey: 24-month results. *J Appl Oral Sci* (2009) 17:307–314. doi: 10.1590/s1678-77572009000400008

143. Frencken JE, van’t Hof MA, Taifour D, Al-Zaher I. Effectiveness of ART and traditional amalgam approach in restoring single-surface cavities in posterior teeth of permanent dentitions in school children after 6.3 years. *Community Dent Oral Epidemiol* (2007) 35:207–214. doi: 10.1111/j.1600-0528.2006.00322.x

144. Baca P, Bravo M, Baca AP, Jiménez A, González-Rodríguez MP. Retention of three fissure sealants and a dentin bonding system used as fissure sealant in caries prevention: 12-month follow-up results. *Med oral patol oral cir bucal* (2007) 12:459–463. https://scielo.isciii.es/scielo.php?script=sci_abstract&pid=S1698-69462007000600010&lng=es&nrm=iso&tlng=en [Accessed November 9, 2025]

145. Ramamurthy P, Rath A, Sidhu P, Fernandes B, Nettem S, Fee PA, Zaror C, Tanya C. Walsh T. Sealants for preventing dental caries in primary teeth. *Cochrane Database Syst Rev* (2022) 2022: doi: 10.1002/14651858.CD012981.pub2

146. Innes NP, Evans DJP, Stirrups DR. The Hall Technique; a randomized controlled clinical trial of a novel method of managing carious primary molars in general dental practice: acceptability of the technique and outcomes at 23 months. *BMC Oral Health* (2007) 7:18. doi: 10.1186/1472-6831-7-18

147. van Gemert-Schriks MCM, van Amerongen WE, ten Cate JM, Aartman IHA. Three-year survival of single- and two-surface ART restorations in a high-caries child population. *Clin Oral Investig* (2007) 11:337–343. doi: 10.1007/s00784-007-0138-8

148. Lo ECM, Holmgren CJ, Hu D, van Palenstein Helderman W. Six-year follow up of atraumatic restorative treatment restorations placed in Chinese school children. *Community Dent Oral Epidemiol* (2007) 35:387–392. doi: 10.1111/j.1600-0528.2006.00342.x

149. Lozano-Chourio MA, Zambrano O, González H, Quero M. Clinical randomized controlled trial of chemomechanical caries removal (Carisolv). *Int J Paediatr Dent* (2006) 16:161–167. doi: 10.1111/j.1365-263X.2006.00719.x

150. Martignon S, Ekstrand KR, Ellwood R. Efficacy of sealing proximal early active lesions: an 18-month clinical study evaluated by conventional and subtraction radiography. *Caries Res* (2006) 40:382–388. doi: 10.1159/000094282

151. Menezes JPDL, Rosenblatt A, Medeiros E. Clinical evaluation of atraumatic restorations in primary molars: a comparison between 2 glass ionomer cements. *J Dent Child (Chic)* (2006) 73:91–97.

152. Frencken JE, Taifour D, van ’t Hof MA. Survival of ART and amalgam restorations in permanent teeth of children after 6.3 years. *J Dent Res* (2006) 85:622–626. doi: 10.1177/154405910608500708

153. Corona S a. M, Borsatto MC, Garcia L, Ramos RP, Palma-Dibb RG. Randomized, controlled trial comparing the retention of a flowable restorative system with a conventional resin sealant: one-year follow up. *Int J Paediatr Dent* (2005) 15:44–50. doi: 10.1111/j.1365-263X.2005.00605.x

154. Gomez SS, Basili CP, Emilson C-G. A 2-year clinical evaluation of sealed noncavitated approximal posterior carious lesions in adolescents. *Clin Oral Investig* (2005) 9:239–243. doi: 10.1007/s00784-005-0010-7

155. van den Dungen GM, Huddleston Slater AE, van Amerongen WE. [ART or conventional restorations? A final evaluation of proximal restorations in deciduous molars]. *Ned Tijdschr Tandheelkd* (2004) 111:345–349.

156. Loh K. An ART technique in the mobile dental squad in Malaysia: a four-year review. *Malaysian Dent J* (2003) 24:95–101.

157. Mandari GJ, Frencken JE, van’t Hof MA. Six-year success rates of occlusal amalgam and glass-ionomer restorations placed using three minimal intervention approaches. *Caries Res* (2003) 37:246–253. doi: 10.1159/000070866

158. Kalf-Scholte SM, van Amerongen WE, Smith AJE, van Haastrecht HJA. Atraumatic restorative treatment (ART): a three-year clinical study in Malawi--comparison of conventional amalgam and ART restorations. *J Public Health Dent* (2003) 63:99–103. doi: 10.1111/j.1752-7325.2003.tb03482.x

159. Rahimtoola S, van Amerongen E. Comparison of two tooth-saving preparation techniques for one-surface cavities. *ASDC J Dent Child* (2002) 69:16–26, 11.

160. Yip H-K, Smales RJ, Yu C, Gao X-J, Deng D-M. Comparison of atraumatic restorative treatment and conventional cavity preparations for glass-ionomer restorations in primary molars: one-year results. *Quintessence Int* (2002) 33:17–21.

161. Yip KHK, Smales RJ, Gao W, Peng D. The effects of two cavity preparation methods on the longevity of glass ionomer cement restorations: an evaluation after 12 months. *J Am Dent Assoc* (2002) 133:744–751; quiz 769. doi: 10.14219/jada.archive.2002.0272

162. Lo EC, Chu CH, Lin HC. A community-based caries control program for pre-school children using topical fluorides: 18-month results. *J Dent Res* (2001) 80:2071–2074. doi: 10.1177/00220345010800120901

163. Yee R. An ART field study in western Nepal. *Int Dent J* (2001) 51:103–108. doi: 10.1002/j.1875-595x.2001.tb00830.x

164. Kikwilu EN, Mandari GJ, Honkala E. Survival of Fuji IX ART fillings in permanent teeth of primary school children in Tanzania. *East Afr Med J* (2001) 78:411–413. doi: 10.4314/eamj.v78i8.8992

165. Lo EC, Holmgren CJ. Provision of Atraumatic Restorative Treatment (ART) restorations to Chinese pre-school children--a 30-month evaluation. *Int J Paediatr Dent* (2001) 11:3–10. doi: 10.1046/j.1365-263x.2001.00232.x

166. Holmgren CJ, Lo EC, Hu D, Wan H. ART restorations and sealants placed in Chinese school children--results after three years. *Community Dent Oral Epidemiol* (2000) 28:314–320. doi: 10.1034/j.1600-0528.2000.280410.x

167. Mickenautsch S, Rudolph MJ, Ogunbodede EO, Frencken JE. The impact of the ART approach on the treatment profile in a mobile dental system (MDS) in South Africa. *Int Dent J* (1999) 49:132–138. doi: 10.1002/j.1875-595x.1999.tb00897.x

168. Luo Y, Wei SH, Fan MW, Lo EC. Clinical investigation of a high-strength glass ionomer restorative used with the ART technique in Wuhan, China: one-year results. *Chin J Dent Res* (1999) 2:73–78.

169. Mertz-Fairhurst EJ, Curtis JW, Ergle JW, Rueggeberg FA, Adair SM. Ultraconservative and cariostatic sealed restorations: results at year 10. *J Am Dent Assoc* (1998) 129:55–66. doi: 10.14219/jada.archive.1998.0022

170. Frencken JE, Makoni F, Sithole WD, Hackenitz E. Three-year survival of one-surface ART restorations and glass-ionomer sealants in a school oral health programme in Zimbabwe. *Caries Res* (1998) 32:119–126. doi: 10.1159/000016441

171. Phantumvanit P, Songpaisan Y, Pilot T, Frencken JE. Atraumatic restorative treatment (ART): a three-year community field trial in Thailand--survival of one-surface restorations in the permanent dentition. *J Public Health Dent* (1996) 56:141–145; discussion 161-163. doi: 10.1111/j.1752-7325.1996.tb02424.x

172. Mertz-Fairhurst EJ, Call-Smith KM, Shuster GS, Williams JE, Davis QB, Smith CD, Bell RA, Sherrer JD, Myers DR, Morse PK. Clinical performance of sealed composite restorations placed over caries compared with sealed and unsealed amalgam restorations. *J Am Dent Assoc* (1987) 115:689–694. doi: 10.14219/jada.archive.1987.0288
