# Supplementary material for: Effect of minimal intervention on carious lesions in primary teeth. An Umbrella review
Source: Front Dent Med. 2026 Jan 12;6:1751752. doi: 10.3389/fdmed.2025.1751752 (PMC12833399; doi:10.3389/fdmed.2025.1751752)
Supplement: Supplementary file 5 [file Table5.docx]

Supplementary Material 4. Assessment of the methodological quality and the quality of the evidence of the included studies

| Authors | Year | AMSTAR – 2 | | | | | | | | | | | | | | | | | Overall confidence |
| --- | --- | --- | --- | --- | --- | --- | --- | --- | --- | --- | --- | --- | --- | --- | --- | --- | --- | --- | --- |
|  |  | 1 | 2* | 3 | 4* | 5 | 6 | 7* | 8 | 9* | 10 | 11* | 12 | | 13* | 14 | 15* | 16 |  |
| Inchingolo et al. (1) | 2025 | Yes | Yes | Yes | Yes partial | Yes | Yes | Yes partial | Yes | Yes | No | No meta-analysis | | | Yes | Yes | No meta-analysis | Yes | High |
| Mohapatra et al. (2) | 2025 | Yes | Yes | Yes | Yes | Yes | Yes | Yes partial | Yes | Yes | Yes | No meta-analysis | | | Yes | No | No meta-analysis | Yes | High |
| Dipalma et al. (3) | 2025 | Yes | Yes | Yes | Yes partial | Yes | Yes | Yes partial | Yes | Yes partial | No | No meta-analysis | | | Yes | No | No meta-analysis | Yes | Moderate |
| Tasleem et al. (4) | 2025 | Yes | Yes | Yes | Yes partial | Yes | Yes | Yes partial | Yes | Yes | No | Yes | Yes | | Yes | Yes | Yes | Yes | High |
| Bukhari et al. (5) | 2025 | Yes | Yes | Yes | Yes | Yes | Yes | Yes partial | Yes | Yes | No | Yes | Yes | | Yes | Yes | Yes | No | Moderate |
| Muntean et al. (6) | 2024 | Yes | Yes | Yes | Yes partial | Yes | Yes | Yes partial | Yes | Yes | No | No meta-analysis | | | Yes | Yes | No meta-analysis | Yes | High |
| Vishwanathaiah et al. (7) | 2024 | Yes | Yes | Yes | Yes | Yes | Yes | Yes partial | Yes | Yes | No | No meta-analysis | | | Yes | Yes | No meta-analysis | Yes | High |
| Alqalaleef et al. (8) | 2024 | Yes | Yes | Yes | Yes partial | Yes | Yes | Yes partial | Yes | Yes | No | Yes | Yes | | Yes | Yes | Yes | Yes | High |
| Inchingolo et al. (9) | 2024 | Yes | Yes | Yes | Yes partial | Yes | Yes | Yes partial | Yes | Yes | No | No meta-analysis | | | Yes | Yes | No meta-analysis | Yes | High |
| Chua et al. (10) | 2023 | Yes | Yes | Yes | Yes | Yes | Yes | Yes partial | Yes | Yes | Yes | Yes | Yes | | Yes | Yes | Yes | Yes | High |
| Cebula et al. (11) | 2023 | Yes | Yes | Yes | Yes partial | Yes | Yes | Yes partial | Yes | Yes | Yes | Yes | Yes | | Yes | Yes | Yes | Yes | High |
| Ramamurthy et al. (12) | 2022 | Yes | Yes | Yes | Yes | Yes | Yes | Yes partial | Yes | Yes | Yes | Yes | Yes | | Yes | Yes | Yes | Yes | High |
| Tedesco et al. (13) | 2022 | Yes | Yes | Yes | Yes | Yes | Yes | Yes partial | Yes | Yes | No | Yes | Yes | | Yes | Yes | Yes | Yes | High |
| Chaudhari et al. (14) | 2022 | Yes | Yes | Yes | Yes partial | Yes | No | No | Yes | Yes | No | No meta-analysis | | | Yes | Yes | No meta-analysis | Yes | Low |
| Hu et al. (15) | 2022 | Yes | Yes | Yes | Yes partial | Yes | Yes | Yes | Yes | Yes | Yes | Yes | | | Yes | Yes | Yes | Yes | Yes |
| Schwendicke et al. (16) | 2021 | Yes | Yes | Yes | Yes | Yes | Yes | Yes | Yes | Yes | Yes | Yes | Yes | | Yes | Yes | Yes | Yes | High |
| Chen et al. (17) | 2021 | Yes | No | Yes | Yes partial | Yes | Yes | Yes partial | Yes | Yes | No | Yes | Yes | | Yes | Yes | Yes | Yes | Low |
| Santamaría et al. (18) | 2020 | Yes | Yes | Yes | Yes partial | Yes | Yes | Yes partial | Yes | Yes | No | No meta-analysis | | | Yes | Yes | No meta-analysis | Yes | High |
| Pagano et al. (19) | 2020 | Yes | No | Yes | Yes | Yes | Yes | Yes partial | Yes | Yes | No | No meta-analysis | | | Yes | Yes | No meta-analysis | Yes | Low |
| Aïem et al. (20) | 2020 | Yes | No | Yes | Yes partial | Yes | Yes | Yes partial | Yes | Yes | No | Yes | Yes | | Yes | Yes | Yes | Yes | Low |
| Jabin et al. (21) | 2020 | Yes | No | Yes | Yes | Yes | Yes | Yes partial | Yes | Yes partial | No | No meta-analysis | | | Yes | Yes | No meta-analysis | Yes | Low |
| Oliveira et al. (22) | 2019 | Yes | Yes | Yes | Yes | Yes | Yes | Yes partial | Yes | Yes | No | Yes | Yes | | Yes | Yes | No | Yes | Low |
| Elrashid et al. (23) | 2019 | Yes | No | Yes | Yes partial | No | No | Yes partial | Yes | Yes | No | Yes | Yes | | Yes | Yes | No | Yes | Critically low |
| Trieu et al. (24) | 2019 | Yes | No | Yes | Yes partial | Yes | Yes | Yes partial | Yes | Yes partial | No | Yes | Yes | | Yes | Yes | Yes | Yes | Low |
| Pedrotti et al. (25) | 2019 | Yes | No | Yes | Yes | Yes | Yes | No | Yes | Yes | No | Yes | Yes | | Yes | Yes | Yes | No | Critically low |
| Li et al. (26) | 2018 | Yes | No | Yes | Yes partial | Yes | Yes | Yes partial | Yes | Yes | No | Yes | Yes | | Yes | Yes | Yes | Yes | Low |
| Deng et al. (27) | 2018 | Yes | No | Yes | Yes partial | Yes | Yes | Yes partial | Yes | Yes | No | Yes | Yes | | Yes | Yes | No | Yes | Critically low |
| Tedesco et al. (28) | 2018 | Yes | Yes | Yes | Yes | Yes | Yes | Yes | Yes | Yes | No | Yes | Yes | | Yes | Yes | Yes | Yes | High |
| Ruengrungsom et al. (29) | 2018 | Yes | No | Yes | No | Yes | Yes | Yes partial | Yes | Yes | No | No meta-analysis | | | No | Yes | No meta-analysis | No | Critically low |
| Dorri et al. (30) | 2017 | Yes | Yes | Yes | Yes | Yes | Yes | Yes | Yes | Yes | Yes | Yes | | Yes | Yes | Yes | Yes | Yes | High |
| Contreras et al. (31) | 2017 | Yes | No | Yes | Yes partial | Yes | Yes | Yes partial | Yes | Yes | No | No meta-analysis | | | Yes | Yes | No meta-analysis | No | Low |
| Papageorgiou et al. (32) | 2017 | Yes | Yes | Yes | Yes | Yes | Yes | Yes | Yes | Yes | No | Yes | | Yes | Yes | Yes | Yes | Yes | High |
| Chibinski et al. (33) | 2017 | Yes | Yes | Yes | Yes | Yes | Yes | Yes | Yes | Yes | No | Yes | | Yes | Yes | Yes | Yes | Yes | High |
| Tedesco et al. (34) | 2017 | Yes | Yes | Yes | Yes | Yes | Yes | Yes | Yes | Yes | No | Yes | | Yes | Yes | Yes | Yes | Yes | High |
| Montedori et al. (35) | 2016 | Yes | Yes | Yes | Yes | Yes | Yes | Yes | Yes | Yes | Yes | Yes | | Yes | Yes | Yes | Yes | Yes | High |
| Duangthip et al. (36) | 2016 | Yes | No | Yes | Yes | Yes | Yes | Yes partial | Yes | Yes | No | No meta-analysis | | | Yes | Yes | No meta-analysis | No | Low |
| Gao et al. (37) | 2016 | Yes | No | Yes | Yes partial | Yes | Yes | Yes partial | Yes | Yes partial | No | Yes | | Yes | Yes | Yes | Yes | Yes | Low |
| Innes et al. (38) | 2015 | Yes | Yes | Yes | Yes | Yes | Yes | Yes | Yes | Yes | Yes | Yes | | Yes | Yes | Yes | Yes | Yes | High |
| Duangthip et al. (39) | 2015 | Yes | No | Yes | Yes | Yes | Yes | Yes partial | Yes | Yes partial | No | No meta-analysis | | | Yes | Yes | No meta-analysis | No | Low |
| Dorri et al. (40) | 2015 | Yes | Yes | Yes | Yes | Yes | Yes | Yes | Yes | Yes | Yes | Yes | | Yes | Yes | Yes | Yes | Yes | High |
| Lai et al. (41) | 2015 | Yes | No | Yes | Yes partial | Yes | No | Yes | Yes | No | No | Yes | | No | No | Yes | No | Yes | Critically low |
| Schwendicke et al. (42) | 2013 | Yes | No | Yes | Yes | Yes | Yes | Yes | Yes | Yes | No | Yes | | Yes | Yes | Yes | Yes | Yes | Low |
| Ricketts et al. (43) | 2013 | Yes | Yes | Yes | Yes | Yes | Yes | Yes | Yes | Yes | Yes | Yes | | Yes | Yes | Yes | Yes | Yes | High |
| Raggio et al. (44) | 2013 | Yes | No | Yes | No | Yes | Yes | Yes partial | Yes | Yes partial | No | Yes | | No | Yes | Yes | Yes | Yes | Critically low |
| Marinho et al. (45) | 2013 | Yes | Yes | Yes | Yes | Yes | Yes | Yes | Yes | Yes | Yes | Yes | | Yes | Yes | Yes | Yes | Yes | High |
| Ferreira et al. (46) | 2012 | Yes | No | Yes | No | Yes | No | No | Yes | Yes partial | No | No meta-analysis | | | Yes | Yes | No meta-analysis | No | Critically low |
| de Amorim et al. (47) | 2012 | Yes | No | Yes | Yes partial | Yes | Yes | Yes | Yes | No | No | Yes | | No | Yes | Yes | No | Yes | Critically low |
| Ricketts et al. (48) | 2006 | Yes | Yes | Yes | Yes | Yes | Yes | Yes | Yes | Yes | Yes | No meta-analysis | | | Yes | Yes | No meta-analysis | Yes | High |
| van 't Hof et al. (49) | 2006 | Yes | No | Yes | No | No | Yes | Yes partial | Yes | No | No | Yes | | No | Yes | Yes | No | No | Critically low |

AMSTAR = A Measurement Tool to Assess Systematic Reviews

1 = Did the research questions and inclusion criteria for the review include the components of PICO?

2 = Did the report of the review contain an explicit statement that the review methods were established prior to the conduct of the review and did the report justify any significant deviations from the protocol?

3 = Did the review authors explain their selection of the study designs for inclusion in the review?

4 = Did the review authors use a comprehensive literature search strategy?

5 = Did the review authors perform study selection in duplicate?

6 = Did the review authors perform data extraction in duplicate?

7 = Did the review authors provide a list of excluded studies and justify the exclusions?

8 = Did the review authors describe the included studies in adequate detail?

9 = Did the review authors use a satisfactory technique for assessing the risk of bias (RoB) in individual studies that were included in the review?

10 = Did the review authors report on the sources of funding for the studies included in the review?

11 = If meta-analysis was performed, did the review authors use appropriate methods for statistical combination of results?

12 = If meta-analysis was performed, did the review authors assess the potential impact of RoB in individual studies on the results of the meta-analysis or other evidence synthesis?

13 = Did the review authors account for RoB in primary studies when interpreting/discussing the results of the review?

14 = Did the review authors provide a satisfactory explanation for, and discussion of, any heterogeneity observed in the results of the review?

15 = If they performed quantitative synthesis did the review authors carry out an adequate investigation of publication bias (small study bias) and discuss its likely impact on the results of the review?

16 = Did the review authors report any potential sources of conflict of interest, including any funding they received for conducting the review?

* = Critical domain

**References**

1. Inchingolo AM, Inchingolo AD, Morolla R, Riccaldo L, Guglielmo M, Palumbo I, Palermo A, Francesco F, Dipalma G. Pre-formed crowns and pediatric dentistry: a systematic review of different techniques of restorations. *J Clin Pediatr Dent* (2025) 49:1–13. doi: 10.22514/jocpd.2025.001

2. Mohapatra S, Mohandas R. Clinical Outcome Success of Silver-Modified Atraumatic Restorative Treatment (SMART) in Treating Children with Dental Caries in Primary Teeth: A Systematic Review. *JHASNU* (2025) 15:4–10. doi: 10.1055/s-0044-1788659

3. Dipalma G, Inchingolo AM, Casamassima L, Nardelli P, Ciccarese D, De Sena P, Inchingolo F, Palermo A, Severino M, Maspero CMN, et al. Effectiveness of Dental Restorative Materials in the Atraumatic Treatment of Carious Primary Teeth in Pediatric Dentistry: A Systematic Review. *Children (Basel)* (2025) 12:511. doi: 10.3390/children12040511

4. Tasleem R, Alqahtani SA, Abogazalah N, Almubarak H, Riaz A, Ali SS, Allana Z. Microinvasive interventions in the management of proximal caries lesions in primary and permanent teeth- systematic review and meta-analysis. *BMC Oral Health* (2025) 25:48. doi: 10.1186/s12903-024-05400-5

5. Bukhari OM. Effectiveness of topical silver diamine fluoride for management of dental caries in children and early adolescents: A systematic review and meta-analysis. *Rom J Oral Rehabil* (2025) 17:975–992. doi: 10.62610/RJOR.2025.2.17.89

6. Muntean A, Mzoughi SM, Pacurar M, Candrea S, Inchingolo AD, Inchingolo AM, Ferrante L, Dipalma G, Inchingolo F, Palermo A, et al. Silver Diamine Fluoride in Pediatric Dentistry: Effectiveness in Preventing and Arresting Dental Caries-A Systematic Review. *Children (Basel)* (2024) 11:499. doi: 10.3390/children11040499

7. Vishwanathaiah S, Maganur PC, Syed AA, Kakti A, Hussain Jaafari AH, Albar DH, Renugalakshmi A, Jeevanandan G, Khurshid Z, Ali Baeshen H, et al. Effectiveness of silver diamine fluoride (SDF) in arresting coronal dental caries in children and adolescents: a systematic review. *J Clin Pediatr Dent* (2024) 48:27–40. doi: 10.22514/jocpd.2024.101

8. Alqalaleef SS, Alnakhli RA, Ezzat Y, AlQadi HI, Aljilani AD, Natto ZS. The role of silver diamine fluoride as dental caries preventive and arresting agent: a systematic review and meta-analysis. *Front Oral Health* (2024) 5:1492762. doi: 10.3389/froh.2024.1492762

9. Inchingolo F, Inchingolo AD, Latini G, Sardano R, Riccaldo L, Mancini A, Palermo A, Inchingolo AM, Dipalma G. Caries in primary molars: is silver diamine fluoride effective in prevention and treatment? A systematic review. *Appl Sci* (2024) 14:2055. doi: 10.3390/app14052055

10. Chua DR, Tan BL, Nazzal H, Srinivasan N, Duggal MS, Tong HJ. Outcomes of preformed metal crowns placed with the conventional and Hall techniques: A systematic review and meta-analysis. *Int J Paediatr Dent* (2023) 33:141–157. doi: 10.1111/ipd.13029

11. Cebula M, Göstemeyer G, Krois J, Pitchika V, Paris S, Schwendicke F, Effenberger S. Resin Infiltration of Non-Cavitated Proximal Caries Lesions in Primary and Permanent Teeth: A Systematic Review and Scenario Analysis of Randomized Controlled Trials. *J Clin Med* (2023) 12:727. doi: 10.3390/jcm12020727

12. Ramamurthy P, Rath A, Sidhu P, Fernandes B, Nettem S, Fee PA, Zaror C, Tanya C. Walsh T. Sealants for preventing dental caries in primary teeth. *Cochrane Database Syst Rev* (2022) 2022: doi: 10.1002/14651858.CD012981.pub2

13. Tedesco TK, Calvo AFB, Pássaro AL, Araujo MP, Ladewig NM, Scarpini S, Lara JS, Braga MM, Gimenez T, Raggio DP. Nonrestorative treatment of initial caries lesion in primary teeth: a systematic review and network meta-analysis. *Acta Odontol Scand* (2022) 80:1–8. doi: 10.1080/00016357.2021.1928748

14. Chaudhari HG, Patil RU, Jathar PN, Jain CA. A systematic review of randomized controlled trials on survival rate of atraumatic restorative treatment compared with conventional treatment on primary dentition. *J Indian Soc Pedod Prev Dent* (2022) 40:112–117. doi: 10.4103/jisppd.jisppd_119_22

15. Hu S, BaniHani A, Nevitt S, Maden M, Santamaria RM, Albadri S. Hall technique for primary teeth: A systematic review and meta-analysis. *Jpn Dent Sci Rev* (2022) 58:286–297. doi: 10.1016/j.jdsr.2022.09.003

16. Schwendicke F, Walsh T, Lamont T, Al-Yaseen W, Bjørndal L, Clarkson JE, Fontana M, Gomez Rossi J, Göstemeyer G, Levey C, et al. Interventions for treating cavitated or dentine carious lesions. *Cochrane Database Syst Rev* (2021) 7:CD013039. doi: 10.1002/14651858.CD013039.pub2

17. Chen Y, Chen D, Lin H. Infiltration and sealing for managing non-cavitated proximal lesions: a systematic review and meta-analysis. *BMC Oral Health* (2021) 21:13. doi: 10.1186/s12903-020-01364-4

18. Santamaría RM, Abudrya MH, Gul G, Mourad MS, Felix Gomez GF, Ferreira Zandona AGF. How to Intervene in the Caries Process: Dentin Caries in Primary Teeth. *Caries Res* (2020) 54:306–323. doi: 10.1159/000508899

19. Pagano S, Lombardo G, Orso M, Abraha I, Capobianco B, Cianetti S. Lasers to prevent dental caries: a systematic review. *BMJ Open* (2020) 10:e038638. doi: 10.1136/bmjopen-2020-038638

20. Aïem E, Joseph C, Garcia A, Smaïl-Faugeron V, Muller-Bolla M. Caries removal strategies for deep carious lesions in primary teeth: Systematic review. *Int J Paediatr Dent* (2020) 30:392–404. doi: 10.1111/ipd.12616

21. Jabin Z, Vishnupriya V, Agarwal N, Nasim I, Jain M, Sharma A. Effect of 38% silver diamine fluoride on control of dental caries in primary dentition: A Systematic review. *J Family Med Prim Care* (2020) 9:1302–1307. doi: 10.4103/jfmpc.jfmpc_1017_19

22. Oliveira BH, Rajendra A, Veitz-Keenan A, Niederman R. The Effect of Silver Diamine Fluoride in Preventing Caries in the Primary Dentition: A Systematic Review and Meta-Analysis. *Caries Res* (2019) 53:24–32. doi: 10.1159/000488686

23. Elrashid AH, Alshaiji BS, Saleh SA, Zada KA, Baseer MA. Efficacy of Resin Infiltrate in Noncavitated Proximal Carious Lesions: A Systematic Review and Meta-Analysis. *J Int Soc Prev Community Dent* (2019) 9:211–218. doi: 10.4103/jispcd.JISPCD_26_19

24. Trieu A, Mohamed A, Lynch E. Silver diamine fluoride versus sodium fluoride for arresting dentine caries in children: a systematic review and meta-analysis. *Sci Rep* (2019) 9:2115. doi: 10.1038/s41598-019-38569-9

25. Pedrotti D, Cavalheiro CP, Casagrande L, de Araújo FB, Pettorossi Imparato JC, de Oliveira Rocha R, Lenzi TL. Does selective carious tissue removal of soft dentin increase the restorative failure risk in primary teeth?: Systematic review and meta-analysis. *J Am Dent Assoc* (2019) 150:582-590.e1. doi: 10.1016/j.adaj.2019.02.018

26. Li T, Zhai X, Song F, Zhu H. Selective versus non-selective removal for dental caries: a systematic review and meta-analysis. *Acta Odontol Scand* (2018) 76:135–140. doi: 10.1080/00016357.2017.1392602

27. Deng Y, Feng G, Hu B, Kuang Y, Song J. Effects of Papacarie on children with dental caries in primary teeth: a systematic review and meta-analysis. *Int J Paediatr Dent* (2018) 28:361–372. doi: 10.1111/ipd.12364

28. Tedesco TK, Gimenez T, Floriano I, Montagner AF, Camargo LB, Calvo AFB, Morimoto S, Raggio DP. Scientific evidence for the management of dentin caries lesions in pediatric dentistry: A systematic review and network meta-analysis. *PLoS One* (2018) 13:e0206296. doi: 10.1371/journal.pone.0206296

29. Ruengrungsom C, Palamara JEA, Burrow MF. Comparison of ART and conventional techniques on clinical performance of glass-ionomer cement restorations in load bearing areas of permanent and primary dentitions: A systematic review. *J Dent* (2018) 78:1–21. doi: 10.1016/j.jdent.2018.07.008

30. Dorri M, Martinez-Zapata MJ, Walsh T, Marinho VC, Sheiham Deceased A, Zaror C. Atraumatic restorative treatment versus conventional restorative treatment for managing dental caries. *Cochrane Database Syst Rev* (2017) 12:CD008072. doi: 10.1002/14651858.CD008072.pub2

31. Contreras V, Toro MJ, Elías-Boneta AR, Encarnación-Burgos A. Effectiveness of silver diamine fluoride in caries prevention and arrest: a systematic literature review. *Gen Dent* (2017) 65:22–29.

32. Papageorgiou SN, Dimitraki D, Kotsanos N, Bekes K, van Waes H. Performance of pit and fissure sealants according to tooth characteristics: A systematic review and meta-analysis. *J Dent* (2017) 66:8–17. doi: 10.1016/j.jdent.2017.08.004

33. Chibinski AC, Wambier LM, Feltrin J, Loguercio AD, Wambier DS, Reis A. Silver Diamine Fluoride Has Efficacy in Controlling Caries Progression in Primary Teeth: A Systematic Review and Meta-Analysis. *Caries Res* (2017) 51:527–541. doi: 10.1159/000478668

34. Tedesco TK, Calvo AFB, Lenzi TL, Hesse D, Guglielmi CAB, Camargo LB, Gimenez T, Braga MM, Raggio DP. ART is an alternative for restoring occlusoproximal cavities in primary teeth - evidence from an updated systematic review and meta-analysis. *Int J Paediatr Dent* (2017) 27:201–209. doi: 10.1111/ipd.12252

35. Montedori A, Abraha I, Orso M, D’Errico PG, Pagano S, Lombardo G. Lasers for caries removal in deciduous and permanent teeth. *Cochrane Database Syst Rev* (2016) 2016: doi: 10.1002/14651858.CD010229.pub2

36. Duangthip D, Jiang M, Chu CH, Lo ECM. Restorative approaches to treat dentin caries in preschool children: systematic review. *Eur J Paediatr Dent* (2016) 17:113–121.

37. Gao SS, Zhao IS, Hiraishi N, Duangthip D, Mei ML, Lo ECM, Chu CH. Clinical Trials of Silver Diamine Fluoride in Arresting Caries among Children: A Systematic Review. *JDR Clin Trans Res* (2016) 1:201–210. doi: 10.1177/2380084416661474

38. Innes NPT, Ricketts D, Chong LY, Keightley AJ, Lamont T, Santamaría RM. Preformed crowns for decayed primary molar teeth. *Cochrane Database Syst Rev* (2015) 2015:CD005512. doi: 10.1002/14651858.CD005512.pub3

39. Duangthip D, Jiang M, Chu CH, Lo ECM. Non-surgical treatment of dentin caries in preschool children--systematic review. *BMC Oral Health* (2015) 15:44. doi: 10.1186/s12903-015-0033-7

40. Dorri M, Dunne SM, Walsh T, Schwendicke F. Micro-invasive interventions for managing proximal dental decay in primary and permanent teeth. *Cochrane Database Syst Rev* (2015) 2015:CD010431. doi: 10.1002/14651858.CD010431.pub2

41. Lai G, Lara Capi C, Cocco F, Cagetti MG, Lingström P, Almhöjd U, Campus G. Comparison of Carisolv system vs traditional rotating instruments for caries removal in the primary dentition: A systematic review and meta-analysis. *Acta Odontol Scand* (2015) 73:569–580. doi: 10.3109/00016357.2015.1023353

42. Schwendicke F, Dörfer C, Paris S. Incomplete Caries Removal: A Systematic Review and Meta-analysis. *J Dent Res* (2013) 92:306–314. doi: 10.1177/0022034513477425

43. Ricketts D, Lamont T, Innes NPT, Kidd E, Clarkson JE. Operative caries management in adults and children. *Cochrane Database Syst Rev* (2013) 28:CD003808. doi: 10.1002/14651858.CD003808.pub3

44. Raggio DP, Hesse D, Lenzi TL, Guglielmi CAB, Braga MM. Is Atraumatic restorative treatment an option for restoring occlusoproximal caries lesions in primary teeth? A systematic review and meta-analysis. *Int J Paediatr Dent* (2013) 23:435–443. doi: 10.1111/ipd.12013

45. Marinho V, Worthington H, Walsh T, Clarkson J. Fluoride varnishes for preventing dental caries in children and adolescents. *Cochrane Database Syst Rev* (2013) 2013:CD002279. doi: 10.1002/14651858.CD002279.pub2

46. Ferreira JMS, Pinheiro SL, Sampaio FC, de Menezes VA. Caries removal in primary teeth--a systematic review. *Quintessence Int* (2012) 43:e9-15.

47. de Amorim RG, Leal SC, Frencken JE. Survival of atraumatic restorative treatment (ART) sealants and restorations: a meta-analysis. *Clin Oral Investig* (2012) 16:429–441. doi: 10.1007/s00784-011-0513-3

48. Ricketts DNJ, Kidd E a. M, Innes N, Clarkson J. Complete or ultraconservative removal of decayed tissue in unfilled teeth. *Cochrane Database Syst Rev* (2006) 19:CD003808. doi: 10.1002/14651858.CD003808.pub2

49. van ’t Hof MA, Frencken JE, van Palenstein Helderman WH, Holmgren CJ. The atraumatic restorative treatment (ART) approach for managing dental caries: a meta-analysis. *Int Dent J* (2006) 56:345–351. doi: 10.1111/j.1875-595x.2006.tb00339.x
